# Design and TCAD analysis of few-layer graphene/ZnO nanowires heterojunction-based photodetector in UV spectral region

**DOI:** 10.1038/s41598-025-92596-3

**Published:** 2025-03-05

**Authors:** Shonak Bansal, Sandeep Kumar, Arpit Jain, Vinita Rohilla, Krishna Prakash, Anupma Gupta, Tanweer Ali, Abdulmajeed M. Alenezi, Mohamed Shabiul Islam, Mohamed S. Soliman, Mohammad Tariqul Islam

**Affiliations:** 1 Department of Electronics and Communication Engineering, Chandigarh University, Gharuan, Punjab India; 2 School of Computer Science and Artificial Intelligence, SR University, Warangal, India; 3 Department of Computer Science and Engineering, Koneru Lakshmaiah Education Foundation Vadeshawaram, A.P Guntur, India; 4 Department of Computer Science and Engineering, Maharaja Surajmal Institute of Technology, C-4 Janakpuri, New Delhi, India; 5 Department of Electronics and Communication, NRI Institute of Technology, Agripalli, Eluru, 521212 AP India; 6 Department of Electronics and Communication Engineering, Saveetha School of Engineering, Saveetha Institute of Medical and Technical Sciences, Thandalam, Chennai, Tamilnadu India; 7 Department of Electronics and Communication Engineering, Manipal Institute of Technology, Manipal Academy of Higher Education, Manipal, 576104 India; 8 Department of Electrical Engineering, Faculty of Engineering, Islamic University of Madinah, Madinah, 42351 Saudi Arabia; 9 Faculty of Engineering (FOE), Multimedia University (MMU), Cyberjaya, 63100 Selangor Malaysia; 10 Department of Electrical Engineering, College of Engineering, Taif University, Taif, 21944 Saudi Arabia; 11 Department of Electrical, Electronic and Systems Engineering, Faculty of Engineering and Built Environment, 43600 UKM Bangi, Selangor Bangi, Malaysia

**Keywords:** External quantum efficiency, Few-layer graphene, Nanowires, Photocurrent responsivity, Photoswitching, ZnO, Nanoscience and technology, Optics and photonics

## Abstract

Graphene and zinc oxide (ZnO) nanowires (NWs)-based photodetectors demonstrate excellent photodetection performance in the ultraviolet (UV) spectrum regime. This paper presents the design and analysis of a heterostructure model of p^+^-few-layer graphene (p^+^-FLG)/n^–^-ZnO NWs-based UV photodetector. The design utilizes the unique properties of few-layer graphene to enhance light absorption and improve photodetector performance. The analysis under both self-biasing and conductive modes of operation reveals that the integrated electric field and the photovoltaic effect at the p⁺-FLG/n⁻-ZnO NWs hetero-interface create a rectifying behavior. The photodetector achieves an external photocurrent responsivity, external quantum efficiency, detectivity, and noise equivalent power of 0.12 A/W, 44.1%, 1.9 × 10^9^ Jones, and 5.6 × 10^–14^ W, respectively, under UV illumination at 350 nm, 0 V bias, and 300 K. Additionally, the photodetector exhibits ultrafast photoswitching rise and fall times of 0.26 ns and a 3-dB cut-off frequency of 1.31 GHz. The comparative analysis with existing photodetectors demonstrates that the proposed model surpasses many in sensitivity, speed, and efficiency. The enhancement of charge collection with the applied reverse-biased voltage results in a response time of 0.16 ns, a peak photocurrent responsivity of 0.2 A/W, a maximum external quantum efficiency of 61%, a peak detectivity of 2.4 × 10^9^ Jones, and minimum noise equivalent power of 4.4 × 10^–14^ W at − 0.5 V. The findings inspire the development of next-generation self-driving, highly efficient, broadband photodetectors, and other economically viable and multifunctional optoelectronic devices.

**Supplementary Information:**

The online version contains supplementary material available at 10.1038/s41598-025-92596-3.

## Introduction

**Fig. 1:**
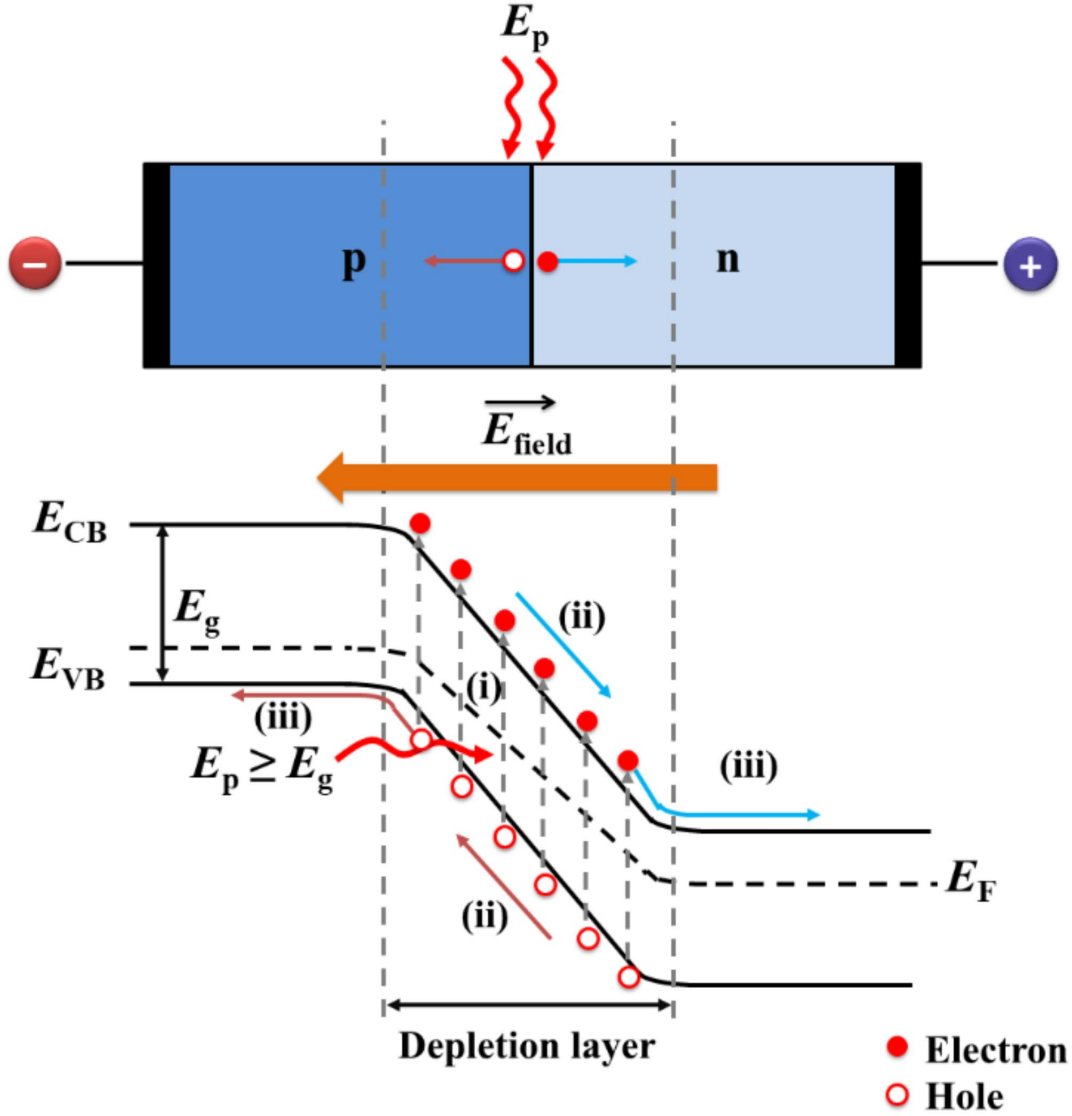
Schematic representation of photodetection. The process involves exciton generation, separation, drift, and charge collection, facilitated by a built-in electric field. The electric field is generated either by the built-in potential of the junction or an external bias supply, enabling efficient photocarrier transport and extraction. Here, *E*_CB_, *E*_VB_, and *E*_F_ represent the conduction band, valence band, and the Fermi level energy, respectively.

Photodetectors, also known as photosensors, play a crucial role in converting incident optical energy to electrical energy across numerous applications, including optical telecommunications, image sensing, remote control, environment monitoring (pollution and ozone), space communication, military surveillance, medical/biological diagnostics, astronomical observations, integrated circuits, missile warning systems, and flame detection^1–6^. The field has seen significant advancements in recent years, driven by innovative materials and nanostructures. Ideal photodetectors aim to achieve miniaturization, low noise, fast response times, high sensitivity (responsivity), wide bandwidth, and high gain-bandwidth products^7,8^. Generally, the operation of photodetectors involves three key steps (Fig. 1): (i) generation of charge carriers (electron-hole pairs) through the absorption of incident light; (ii) separation and transport of photo-induced carriers; and (iii) extraction of charge carriers at the electrodes to produce a measurable electrical signal.

When photons with energy (*E*_p_) exceeding the material’s bandgap (*E*_g_) strike the photodetector, they excite electrons from the valence band to the conduction band, creating electron-hole pairs. In devices with a p-n or Schottky junction, a built-in electric field $\:\left(\overrightarrow{{E}_{\text{field}}}\right)$ efficiently separates these carriers, driving electrons toward the n-type region and holes toward the p-type region, resulting in photocurrent generation. An external bias voltage enhances this process in photoconductive mode, improving carrier separation and collection. Conversely, the device operates without external power in photovoltaic mode, relying solely on the built-in electric field to separate and transport the carriers^9^.

Several large-bandgap materials including zinc oxide (ZnO), gallium nitride (GaN), indium tin oxide (ITO), titanium dioxide (TiO_2_), and tin oxide (SnO) have been investigated for photodetection^4,10^. Among these materials, ZnO, intrinsically an n-type material, has emerged as a particularly suitable choice for room-temperature ultraviolet (UV) detection due to its direct band gap (3.37 eV), low cost, moderate exciton binding energy (60 meV) which enhances UV light absorption and exciton stability at room temperature, superior UV absorption with increased transparency in the visible spectrum regime, abundant nanostructure formation capability, fast switching time, and excellent thermal, mechanical, and chemical stability at room temperature^2,3,5^.

Conventional photodetectors require an external bias voltage to generate photocurrent, which limits their performance in terms of circuit noise, dark current density, and power consumption^2^. In contrast, self-biasing (or self-powered or self-driving) photodetectors can sense incident optical energy without any external bias voltage, utilizing either the photovoltaic effect in p-n junctions or Schottky junction-based mechanisms. These self-driving photodetectors have found potential applications in gas sensors, solar cells, optical switches, remote sensing, and wireless communication systems.

The field of photodetection has seen significant advancements in recent years, driven by innovative materials and nanostructures. Efforts have focused on enhancing responsivity, response speed, spectral sensitivity, and stability, with researchers exploring diverse materials and architectures. Advances in photodetector technologies have included diverse materials such as quantum wells^11^, quantum dots^12^, organic perovskites^13^, and inorganic nanomembranes^14^, yet significant challenges remain in achieving high UV responsivity, fast response speeds, and stability^15–17^.

Among recent advancements, TiO_2_ nanorod-based devices have demonstrated remarkable UV photodetection performance, offering high photoresponsivity and rapid response times due to optimized nanostructured interfaces and the wide bandgap of TiO_2_^18^. Similarly, ZnO nanoparticle-decorated CsPbBr_3_ quantum dot heterostructures have exhibited self-powered operation with enhanced photoresponsivity and stability over seven months, highlighting their potential for practical applications^19^. Furthermore, Al/ZnO quantum dot heterostructures have achieved significant enhancement in photocurrent with a photocurrent responsivity of 11.98 A/W, utilizing plasmonic effects and optimized carrier transport through self-assembled Al nanostructures^20^. Biohybrid approaches have also shown promise, as evidenced by ZnO nanorod arrays combined with biosynthetic pyomelanin, achieving a photocurrent responsivity of 3.91 × 10³ A/W, demonstrating excellent long-term stability^21^. Moreover, the heterojunction of ZnO/Ga_2_O_3_ has shown substantial promise for UV photodetectors, offering enhanced performance and suitability for portable and cost-effective applications^22^. Flexible self-powered photodetectors, such as those utilizing ZnO-amorphous Ga_2_O_3_ core-shell heterojunction microwires, have achieved commendable rectifying behavior, a peak photocurrent responsivity of about 0.13 A/W at 265 nm, and consistent performance under various bending conditions, thereby enabling potential applications in wearable and portable electronics^23^.

Despite these advancements, challenges persist in optimizing photodetector stability, minimizing dark current, and achieving high photocurrent responsivity across a broad spectral range. In response, researchers have increasingly turned to the heterojunctions of ZnO with two-dimensional (2D) materials to address these limitations. For example, incorporating graphene layers into an n-Si/n-ZnO heterostructure has significantly improved photoelectrochemical activity by leveraging graphene’s superior charge transport properties and light absorption capabilities^17^. Similarly, Ghanbari et al.^2^ developed a self-biased broadband photodetector using p-WSe_2_/n-ZnO (2D/3D) heterojunction, achieving a quantum efficiency of 18% and a photocurrent responsivity of ∼0.045 A/W at a 300 nm wavelength. This performance is 10 times higher than the ZnO/(Cu-doped ZnO) core/shell nanorods-based photodetector^24^. Additionally, an n-MoSe_2_/n-ZnO (2D/three-dimensional (3D)) heterojunction photodetector^25^ was proposed with a response time of 40 µs.

Compared to traditional planar p-n junction structures, one-dimensional (1D) nanostructures, including nanowires, nanopillars, nanotubes, nanocages, nanobelts, and nanopropellers have been proposed and examined to enhance photodetection response due to their conductive channels, well-modulated electronic transport, and volumetric light absorption properties. Vertically aligned nanowires (NWs) have gained particular attention as a 1D nanostructure due to their increased surface area for light harvesting, enhanced absorption, reduced semiconductor material requirements, fast charge transport, and improved charge collection^3,26^. Unlike their planar counterparts, NW architecture eliminates the need for anti-reflection layers due to reduced light reflection^26^. Fabrication of NWs can be achieved through various experimental methods involving chemical and physical deposition techniques^27^. In NW architecture design, the photogenerated carriers move a very short path before the external electrodes are collected, leading to better charge collection and efficiency.

The NWs-based photodetectors can be applied as single-photon detectors, optical intrachip interconnects, and image sensors^28^. ZnO NWs are preferred for UV photodetectors due to their high sensitivity, large surface-to-volume ratio, better absorption, efficient electron transport, and enhanced photoresponse under UV illumination^3,29^. It has been proposed that a heterojunction of 2D material with ZnO NWs significantly improves the photodetection performance in photodetectors compared to ZnO NWs alone^2,3,10,30^.

In addition to these advancements, perovskite-based photodetectors have emerged as promising candidates due to their excellent light absorption, tunable bandgap, and cost-effective fabrication. The heterostructure of ZnO NWs with perovskite demonstrates superior performance across a broad detection range, spanning from UV to visible, compared to standalone ZnO or perovskite photodetectors^31^. Bio-inspired photodetectors, such as polarization-sensitive designs inspired by desert ant compound eyes, have demonstrated exceptional applications in bionic navigation, stress visualization, and medical diagnostics^32^. Integrating biocompatible ZnO NWs into photodetectors enables real-time monitoring of physiological parameters, such as glucose levels and oxygen saturation, while facilitating UV exposure monitoring on the skin to help prevent conditions like melanoma. When incorporated into wearable devices, these photodetectors provide real-time feedback, allowing timely interventions and enhanced skin protection strategies^33^. Visible-blind photodetectors, particularly those based on wide-bandgap materials, have also demonstrated excellent potential for detecting UV radiation while minimizing interference from visible light. The high sensitivity of ZnO NW photodetectors to UV light, combined with their visible-blindness, makes them ideal for integration into a wide range of biomedical devices^33^. Flexible photodetectors, leveraging ultrathin films and nanostructures, have enabled wearable and portable devices suitable for real-time monitoring. ZnO NWs, with their outstanding electronic properties and mechanical flexibility, are particularly well-suited for flexible and implantable electronics, further advancing the capabilities of flexible photodetectors. These photodetectors also exhibit exceptional stretchability, maintaining consistent performance even under significant mechanical strain, making them ideal for wearable and flexible electronics applications^33^. Moreover, innovations in UV photodetectors, including novel material engineering and heterostructure designs, have broadened their applicability in environmental monitoring, communication, and security^3^.

Among the various 2D materials studied in recent years, graphene heterojunction with different semiconductor elements facilitates high-performance photodetection in self-biasing mode due to the strong electric field at the heterojunction^34^. Graphene provides high sensitivity, high carrier transport mobility, low resistivity, high current carrying ability, wide-range absorption spectral response, mechanical flexibility, and a tunable Fermi level^10,35,36^. The heterojunction of graphene provides a high electric field that quickly separates the photogenerated carriers without the need for external bias voltage, thus offering high performance and a self-biasing mode of operation^34^. Graphene works as a light anti-reflection coating, aiming to reduce light reflection^26^ while facilitating efficient carrier separation through its adjustable Fermi level^35^. Graphene can also be used as a growth template^10,37^, a Schottky contact^38^, and even just a transparent electrode^39^. Therefore, graphene is a suitable material choice for enhanced photodetection performance in heterojunction-based photodetectors in broad-spectrum regimes^10^. However, single-layer graphene (SLG)-based photodetectors face challenges due to their null band gap and small optical absorption of about 2.3%, resulting in large leakage current density and low photocurrent responsivity (< 1 mA/W) at room temperature^40,41^.

Among various graphene/NWs configurations^37,38,42–48^, graphene/ZnO NWs-based photodetectors have attracted major attention due to their unique properties, including 1D carrier transport capability, good crystal quality, superior optoelectronic properties, fast response, and high sensitivity^49,50^. Significant progress in this field has been made through several notable studies. Fu et al.^38^ demonstrated high photosensitivity using a single ZnO NW sandwiched between graphene layers. Nie et al.^51^ achieved 113 A/W photocurrent responsivity by transferring graphene onto ZnO nanorod arrays, while Zhang et al.^39^ modified interface properties using multiple graphene sheets, resulting in exceptional UV responsivity. Boruah et al.^52^ observed improved performance by sandwiching ZnO NWs between graphene layers, achieving a quantum efficiency of 7845.54% and a photocurrent responsivity of 23 A/W under UV illumination. Xu et al.^37^ reported enhanced crystal quality and responsivity of 188 A/W at 390 nm using a hydrothermal technique for ZnO NW growth on graphene. Additionally, Bai et al.^53^ reported improved light absorption using a graphene layer between ZnO NW arrays and cuprous oxide, though responsivity remains an area for improvement. Wang et al.^54^ further introduced a flexible photodetector using a graphene/ZnO NWs heterostructure, demonstrating minimal response variation under strain but facing performance challenges due to interfacial issues. Chakraborty et al.^55^ showcased UV detection with ZnO NWs layered with TiO_2_-graphene oxide, achieving a photocurrent responsivity of 13.52 A/W. These studies collectively demonstrate both the progress and ongoing challenges in this field.

Photodetectors constructed using SLG and ZnO NWs often experience challenges, such as high dark current density and poor photodetection response. These limitations can be attributed to the absence of bandgap in single-layer graphene and the relatively limited light absorption capabilities of single-layered graphene. To address limitations associated with SLG-based photodetectors, chemically doped graphene layers have been explored to enhance light absorption and reduce dark current density^40,56–59^. The introduction of p-type semiconductor doping in graphene layers shifts the Fermi level away from the conduction band and closer to the valence band^57^, resulting in increased barrier height and an enhanced electric field at the junction. This enhanced field improves carrier generation, drift velocity, and system efficiency^40,57^.

Based on the above discussion, this paper proposes the heterostructure of highly p-type (p^+^) doped few-layer graphene (FLG) and lightly n-type (n^–^) doped ZnO NWs-based UV photodetector under both self-biasing and conductive modes of operation. The Silvaco TCAD software is used in a 3D environment to design and evaluate the optoelectronic performance parameters of the p^+^-FLG/n^–^-ZnO NWs-based UV photodetector at 300 K. The exploration of FLG/ZnO NWs heterojunction structures has not garnered as much academic attention compared to the graphene/ZnO heterojunction in recent years. This current research aims to examine how the number of graphene layers affects the photodetection capabilities of the device.

With a large number of graphene layers, the light absorption capability of FLG is improved, resulting in enhanced photodetection in the proposed UV photodetector. This also reduces dark current, a crucial factor for improving the signal-to-noise ratio and overall detectivity of the device. Moreover, the obtained optoelectronic properties of the proposed heterojunction UV photodetector are further validated by analytical modeling. The obtained results suggest that the p^+^-FLG/n^–^-ZnO NWs-based heterojunction photodetector is an excellent device for the development of self-driving, highly efficient, broadband photodetectors, and other cost-effective multifunctional optoelectronic devices.

**Fig. 2:**
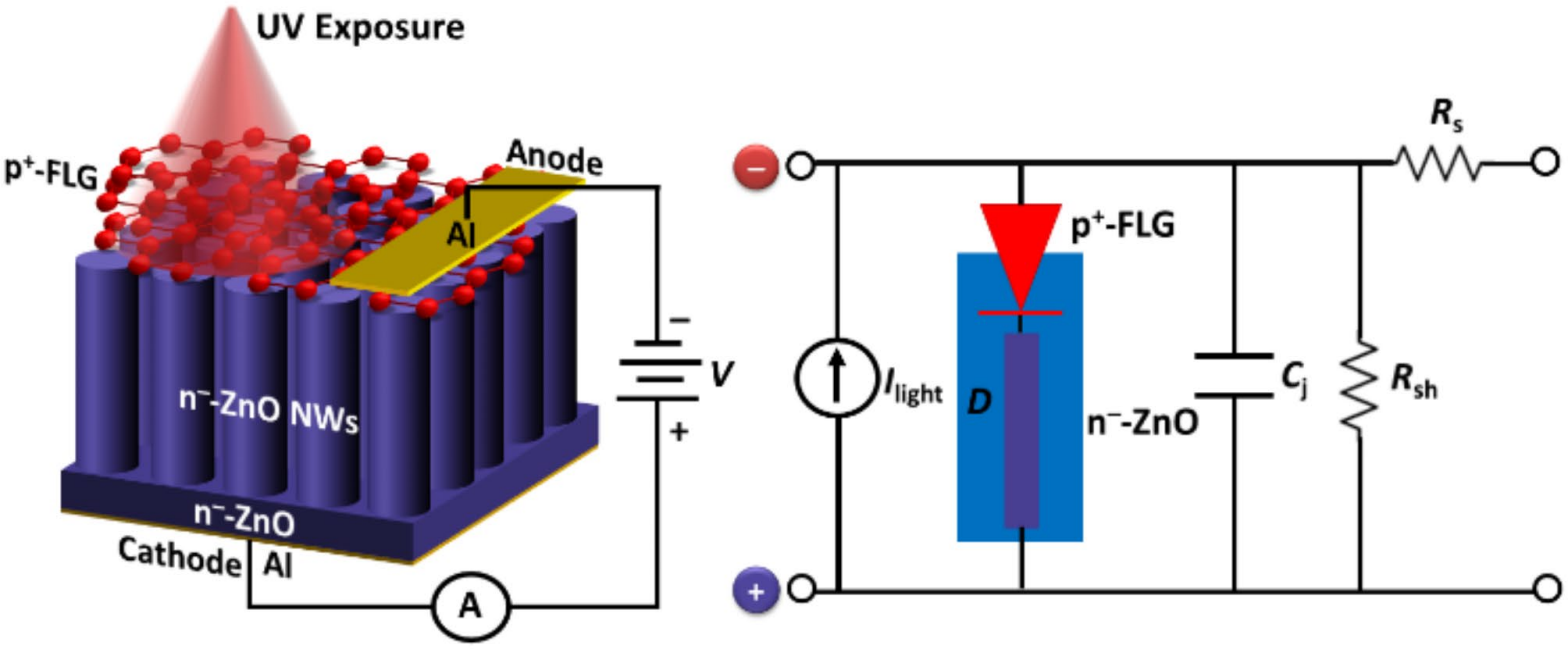
Schematic and equivalent electrical circuit illustration of p^+^-FLG/n^–^-ZnO NWs based heterojunction UV photodetector. The equivalent circuit diagram includes a photodiode (*D*) representing the p^+^-FLG/n^–^-ZnO NW heterojunction, series resistance (*R*_s_), shunt resistance (*R*_sh_), junction capacitance (*C*_j_), and a light-dependent current source (*I*_light_) placed in parallel with the photodiode, modeling the electrical characteristics of the photodetector.

## Proposed methodology

Manufacturing photodetectors is a highly intricate task typically performed by numerous humanoid teams and is responsible for theoretical modeling (including material parameters and numerical simulations), design, epitaxial growth, processing, and characterization. This work focuses on theoretical modeling and mainly concerns the optoelectronic characteristics of the FLG/ZnO NWs-based heterojunction UV photodetector using Silvaco device simulation software. Figure 2 presents the cross-sectional view of the p^+^-FLG/n^–^-ZnO NWs-based heterojunction UV photodetector, accurately representing the structure used in the simulation. The schematic illustrates the layered structure from bottom to top: the n^–^-ZnO seed layer, vertically aligned n^–^-ZnO NWs, and the top p^+^-FLG layer. The photodetector is top illuminated within the UV spectrum regime (λ < 380 nm) having an incident power density *P*_in_ of 1 W/cm^2^.

Upon illumination, n^–^-ZnO NWs act as the primary light absorber in the UV spectrum, while the p^+^-FLG layer enhances carrier separation and transport at the heterojunction. The interface between p^+^-FLG and n^–^-ZnO NWs forms a critical heterojunction, modeled in the simulation as an abrupt junction with appropriate band alignment. At this interface, a depletion region is created due to the difference in Fermi levels of the two materials. This results in a strong built-in electric field $\:\overrightarrow{{E}_{\text{field}}}$ at the hetero-interface, which is crucial for the device’s self-biasing operation.

When electron-hole pairs are generated predominantly in the n^–^-ZnO NWs under UV illumination, the $\:\overrightarrow{{E}_{\text{field}}}$ at the heterojunction separates these charges. Electrons are swept towards the n^–^-ZnO NWs, while holes remain in the p^+^-FLG layer. This efficient charge separation at the heterojunction, driven by the built-in electric field, plays a crucial role in generating photocurrent, enabling self-biasing operation.

Beyond their role in forming the heterojunction with p^+^-FLG, the n^–^-ZnO NWs are engineered to maximize UV light absorption and carrier generation. To optimize the performance of the photodetector, the ZnO NWs are designed as a vertically aligned array, with each nanowire having a precisely controlled diameter of 200 nm and a length of 1500 nm, serving as the active photodetecting material. These dimensions are carefully chosen to balance several critical factors. The 1500 nm length provides sufficient material volume for efficient light absorption, particularly for wavelengths near ZnO’s band edge. The 200 nm diameter ensures photogenerated carriers can reach the nanowire surface before recombination, optimizing charge collection efficiency. The nanowires’ high aspect ratio (7.5:1) significantly increases the effective surface area for light interaction and charge separation at the ZnO/FLG interface. These dimensions also maintain a balance between height for absorption and width for mechanical stability of the nanowire array. The nanowires are spaced 20 nm apart, ensuring optimal packing density for increased absorption and efficient charge transport, covering a total active area of 1.08 × 1.08 µm^2^. This design maximizes the ZnO/FLG interface area while maintaining structural integrity, thereby enhancing the overall device performance.

To ensure uniform growth and alignment of the ZnO NWs, a thin ZnO seed layer of 10 nm is incorporated at the base as shown in Fig. 2. This seed layer, having the same doping concentrations as the ZnO NWs, provides a continuous path for carrier collection and improves the electrical contact to the n-type region of the device. Experimentally, the ZnO NWs can be grown using a hydrothermal method on top of this seed layer, which is first deposited by atomic layer deposition^53^. In this work, the ZnO NWs are modeled as discrete structures to accurately capture their geometry and optical properties, which are vital for understanding their contribution to the device’s high photodetection efficiency. The seed layer, however, is modeled as a continuous film with properties matching those of bulk ZnO. This detailed design and modeling approach of the ZnO NWs enables precise control over the photodetector’s optical and electrical properties, facilitating high-performance photodetection across a spectral range.

The lightly n-doped ZnO NWs are interfaced with the highly p-doped FLG layer, designed to have a bandgap of approximately 250 meV and a thickness of 2 nm (~ 6 layers of graphene). This bandgap value is chosen based on reported behavior in heavily doped FLG^3,40,55–61^, where doping can induce a tunable bandgap due to Fermi level shifts and symmetry-breaking effects, especially at high doping concentrations. The p-type doping of the graphene can be realized through the chemical doping of nitric acid (HNO_3_), thionyl chloride (SOCl_2_), gold trichloride (AuCl_3_), ferric chloride (FeCl_3_), and niobium chloride (NbCl_5_) via the chemical vapor deposition (CVD) procedure^40,57^. This careful integration of n^–^-ZnO NWs with the p^+^-FLG layer allows for precise control over the photodetector’s optical and electrical properties, enabling high-performance photodetection.

The resistive electrical electrodes of aluminum (Al) are placed on the top and bottom surfaces of the structure as an anode and cathode, respectively, to collect the carriers. In real devices, the graphene is directly grown on a copper foil as the substrate via CVD method deprived of any impurity mixing problem^62^. In the proposed p^+^-FLG/n^–^-ZnO NWs-based heterostructure, the ZnO NWs can be synthesized either by the thermal-oxidation process of magnetron sputtered Zinc layer over a Si surface^63^ or through the thermal-oxidation process of a metallic Zinc layer on a glass substrate^64^. Other methods of ZnO NWs synthesis include CVD^65^, chemical bath deposition^66^, molecular beam epitaxy^67^, and electrochemical deposition^68^. As shown in Fig. 2, the electrical equivalent circuit for the proposed heterojunction UV photodetector consists of an individual ZnO NW inserted between the top and bottom layers of the asymmetric diode. The equivalent circuit model of the FLG/ZnO NWs-based UV photodetector incorporates essential components that represent its physical characteristics and behavior. A photodiode (*D*) represents the p^+^-FLG/n^–^-ZnO NW heterojunction, responsible for the device’s rectifying behavior and photoresponse, generating photocurrent when exposed to UV light. The series resistance (*R*_s_) accounts for resistive losses within the device, including contributions from the graphene layer and ZnO NWs, influencing overall efficiency and response time. A shunt resistance (*R*_sh_) models leakage paths and junction imperfections, with higher values indicating lower leakage currents and improved performance. The junction capacitance (*C*_j_) represents the heterojunction’s capacitance, affecting response speed and frequency characteristics. A light-dependent current source (*I*_light_), placed parallel to the photodiode, simulates the photocurrent generated when UV light is incident on the ZnO NWs, with its magnitude proportional to the incident light intensity. This circuit representation enables analysis of the photodetector’s electrical characteristics and performance under various operating conditions, providing insights into its sensitivity, response time, and overall efficiency.

In this study, consistent doping is used across all areas. It is found that with the increase in the doping concentration, the efficiency of the photodetector reduces^69,70^. This is because when the doping concentration increases, the width of the depletion region decreases, reducing the active volume available for photogeneration. A narrower depletion region limits the generation and separation of photogenerated carriers, negatively impacting the photodetector’s photoresponse and overall efficiency^58,69^. Moreover, higher doping concentrations lead to more carrier recombination due to enhanced impurity and defect densities, which reduces efficiency. While increased doping lowers the material’s resistivity^70^, this advantage is negated by two major drawbacks: narrowing the depletion width and increasing recombination rates. Therefore, an optimized doping concentration of 2 × 10^22^ cm^–3^ for p^+^-FLG^3,40,71^ and 1 × 10^16^ cm^–3^ for n^–^-ZnO^27^ is considered in this work.

This work demonstrates the design and theoretical modeling of the p^+^-FLG/n^–^-ZnO NWs-based heterostructure, performed utilizing Silvaco’s DEVEDIT and ATLAS device simulator platforms. The device structure was carefully constructed within DEVEDIT, followed by extensive simulations in ATLAS to evaluate and examine the performance characteristics of the proposed photodetector. The Silvaco ATLAS device simulator uses a finite element method to find the optoelectronic characteristics of the photodetector. The electrical behavior of the proposed photodetector is analyzed using a fundamental set of semiconductor equations. These equations, derived from the continuity and Poisson equations for electrons and holes, provide a theoretical understanding of the device’s operation^72^

1 $$\:\frac{\partial\:n}{\partial\:t}=\frac{1}{q}\nabla\:.{J}_{\text{n}}+{G}_{\text{n}}-{R}_{\text{n}}$$

2 $$\:\frac{\partial\:p}{\partial\:t}=-\frac{1}{q}\nabla\:.{J}_{\text{p}}+{G}_{\text{p}}-{R}_{\text{p}}$$

3 $$\:{\nabla\:}^{2}V=-\frac{\rho\:}{\epsilon\:}$$

where *q* is the electronic charge, *n* and *p* represent the equilibrium electron and hole concentration, respectively; whereas *J*_n_ and *J*_p_ denote the electron and hole current density, respectively. The generation rate of electron and hole is depicted by the symbols *G*_n_ and *G*_p_, respectively; *R*_n_ and *R*_p_ denote the recombination rates for the electron and hole, respectively. *V* is the electrostatic potential; *ρ* is the space charge density; and *ε* is the permittivity.

By default, the Silvaco ATLAS device simulator solves both the continuity equations, but as per the requirement, the continuity equations can be solved either for electrons and/or holes by using a method statement. In semiconductor materials, drift-diffusion technology is the commonly used method for the simulation of carrier transport. The conventional drift-diffusion technology is approximated by the Boltzmann-transport-theory and current-density-equation. It is the function of carrier concentrations (*n* and *p*), electric field $\:\left(E=-\nabla\:V\right)$, and drift-diffusion components^72^

4 $$\:{J}_{\text{n}}=q{\mu\:}_{\text{n}}nE+q{D}_{\text{n}}\nabla\:n$$

5 $$\:{J}_{\text{p}}=q{\mu\:}_{\text{p}}pE-q{D}_{\text{p}}\nabla\:p$$

where electron and hole mobilities dependent on doping and temperature are represented by *µ*_n_ and *µ*_p_, respectively. The electron diffusion coefficient *D*_n_ and hole diffusion coefficient *D*_p_ are approximated as^72^

6 $$\:{D}_{\text{n}}=\frac{{\mu\:}_{\text{n}}{k}_{\text{B}}T}{q}\:\text{c}\text{m}^2/\text{s}$$

7 $$\:{D}_{\text{p}}=\frac{{\mu\:}_{\text{p}}{k}_{\text{B}}T}{q}\text{c}\text{m}^2/\text{s}$$

here *k*_B_ and *T* correspond to the Boltzmann’s constant and temperature, respectively.

To analyze the optoelectronic properties, the carrier transport diffusion, Poisson, and continuity equations based on Boltzmann’s transport model are solved with suitable boundary conditions by using Newton’s iterative numerical method^72^. To characterize the carrier lifetime and the dark current density in the proposed UV photodetector, Shockley-Read-Hall (SRH), Auger, and optical (band-to-band) recombination rates are considered and are given as^72^

8 $$\:{R}_{\text{SRH}}=\frac{pn-{n}_{\text{i}}^{2}}{{\tau\:}_{\text{p}}\left[n+{n}_{\text{i}}\text{exp}\left({E}_{\text{t}}/{k}_{\text{B}}T\right)\right]+{\tau\:}_{\text{n}}\left[p+{n}_{\text{i}}\text{exp}\left(-{E}_{\text{t}}/{k}_{\text{B}}T\right)\right]}$$

9 $$\:{R}_{\text{Auger}}={C}_{\text{n}}\left(p{n}^{2}-n{n}_{\text{i}}^{2}\right)+{C}_{\text{p}}\left({p}^{2}n-p{n}_{\text{i}}^{2}\right)$$

10 $$\:{R}_{\text{np}}^{\text{Optical}}={C}_{\text{c}}^{\text{OPT}}\left(pn-{n}_{\text{i}}^{2}\right)$$

where *τ*_p_ and *τ*_n_ depict the SRH lifetimes of holes and electrons, respectively. The position of the trap levels in the bandgap is indicated by *E*_t_. *C*_n_ and *C*_p_ are the Auger coefficient of electrons and holes, respectively, and $\:{C}_{\text{c}}^{\text{OPT}}$ denotes the capture rate of carriers.

In this work, the FLG is considered 3D in nature similar to previous works demonstrating the integration of multilayer graphene with other semiconductor materials which couples 3D equations to 2D transport equations satisfying the need to have Fermi wavelength $\:\left({\lambda}_{\text{F}} \: = \: 2 \times1{\text{0}}^{\text{7}}\sqrt{\frac{\pi}{n}}\:\:\text{(nm)}\right)$ smaller than the considered thickness of graphene^40,57,71^. The *λ*_F_ of p^+^-FLG is found to be ~ 0.56 nm for the sheet carrier density (*n*) of 4 × 10^15^ cm^–2^. Since the electrons in graphene have lower effective mass than the holes^73^, therefore electron mobility of graphene is always higher than the hole’s mobility. Table 1 presents the material parameters used for the simulation, adapted from previously reported theoretical and experimental works^3,27,40,57,59,70–72,74–80^. This study primarily focuses on the 5 × 5 ZnO NW array to evaluate its optoelectronic performance in the UV region. Additionally, simulations comparing photodetectors with 2 × 2, 4 × 4, and 5 × 5 ZnO NW arrays were conducted to investigate the effect of nanowire density on key performance parameters. These results, along with additional simulations examining structural optimization, are provided in the supplementary information for deeper insights into device performance and optimization strategies.

**Table 1:** The parameters optimized for the photodetector design at 300 K.

Parameters and units	*p*^+^-FLG	*n*^–^-ZnO
Energy bandgap (*E*_g_) (eV)	0.25	3.37
Affinity (*χ*) (eV)	4.8	4.5
Permittivity (*ε*_r_)	3.3	8.5
Intrinsic carrier concentration (*n*_i_) (cm^–3^)	3.18 × 10^15^ (cal.)	8.26 × 10^–10^ (cal.)
Effective density in the conduction band (*N*_CB_) (cm^–3^)	4 × 10^17^	4 × 10^18^
Effective band density in the valence band (*N*_VB_) (cm^–3^)	4 × 10^17^	7 × 10^19^
Doping concentration (cm^–3^)	2 × 10^22^	1 × 10^16^
Hole lifetime (*τ*_p_) (s)	5 × 10^–12^	1 × 10^–9^
Electron lifetime (*τ*_n_) (s)	5 × 10^–12^	1 × 10^–9^
Hole mobility (*µ*_p_) (cm^2^/Vs)	400	10
Electron mobility (*µ*_n_) (cm^2^/Vs)	40,000	60

**Fig. 3:**
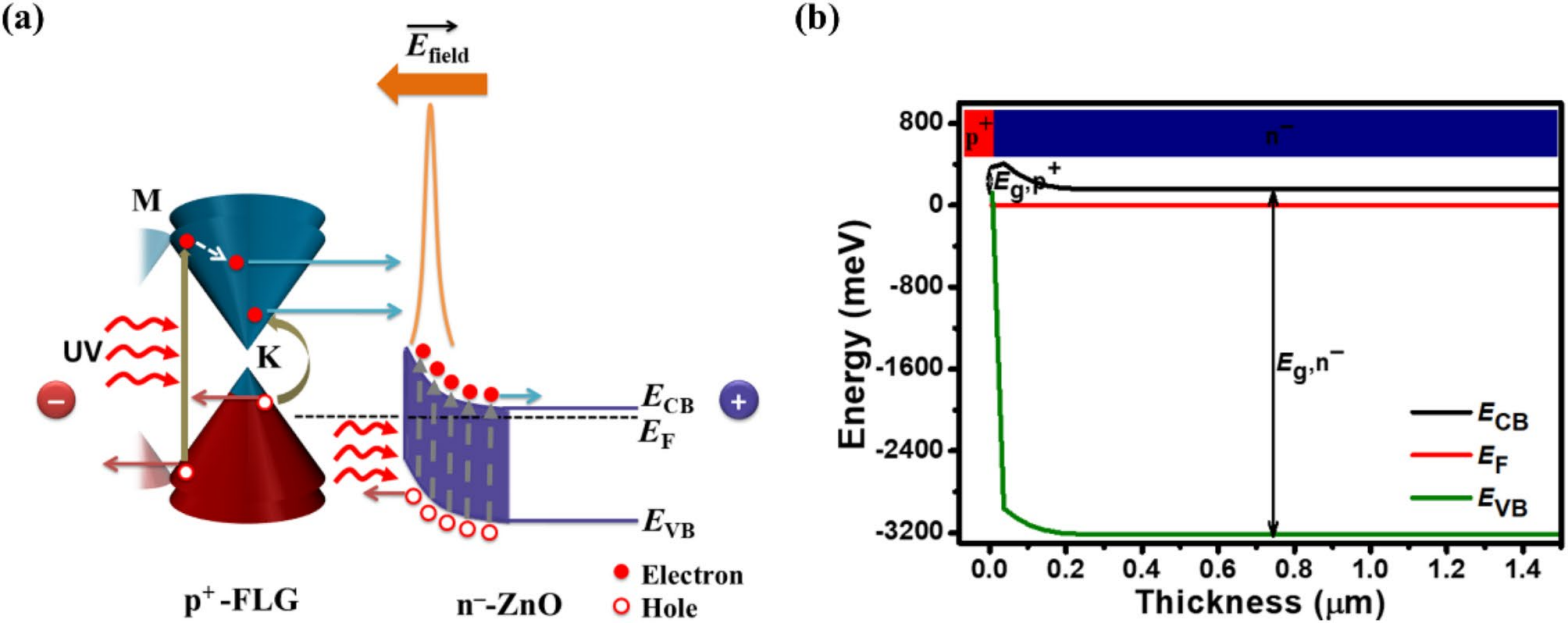
Schematic of the energy bandgap diagram and electric field profile for p^+^-FLG/n^–^-ZnO NWs heterojunction-based UV photodetector. **(a)** Illustration under illumination and biased conditions. **(b)** Simulated energy bandgap diagram under dark conditions in self-biasing mode. *E*_g_,p^+^ and *E*_g_,n^–^ denotes the bandgap of p^+^-FLG and n^–^-ZnO, respectively.

## Results and discussions

The electric field profile and energy bandgap diagram of p^+^-FLG/n^–^-ZnO NWs-based photodetector are depicted in Fig. 3(a). The different work functions of graphene and ZnO result in the formation of a junction barrier at the p^+^-FLG/n^–^-ZnO hetero-interface. By utilizing multiple graphene layers and doping it with p-type dopants, the energy of Fermi level in graphene shifts towards the valence band^57^, leading to an increased barrier height and a significant built-in electric field $\:\overrightarrow{{E}_{\text{field}}}$.

Under equilibrium (dark) conditions, the joining of p^+^-FLG and n^–^-ZnO NWs causes the majority charge carriers to diffuse across the interface due to the concentration gradient. Holes from p^+^-FLG diffuse into the n^–^-ZnO NWs, while electrons from n^–^-ZnO NWs diffuse into the p^+^-FLG. This diffusion continues until the Fermi levels of both materials align, creating a new equilibrium state characterized by band bending at the interface. This band bending forms a potential barrier crucial for the photodetector’s operation.

The depletion region formed at the hetero-interface is characterized by a strong built-in electric field $\:\overrightarrow{{E}_{\text{field}}}$, which is crucial for separating photogenerated electron-hole pairs under illumination. When UV light strikes the device, it generates electron-hole pairs in the p^+^-FLG layer. The $\:\overrightarrow{{E}_{\text{field}}}$ at the heterojunction efficiently drives electrons toward the n^–^-ZnO NWs, while holes remain in the p^+^-FLG layer. This effective charge separation results in photocurrent generation, enabling the photodetector to operate without an external bias.

The width of the depletion region, which influences photogeneration capacity, is dependent on the doping concentrations of the materials. While lower doping levels generally increase the width of the depletion region, providing more space for photogeneration, there is a trade-off: overly low doping can reduce carrier density, potentially limiting device performance. Therefore, the doping concentrations used in this study were optimized to achieve a balance between depletion width and carrier availability. These optimized doping levels are presented in Table 1, which demonstrates how the chosen parameters contribute to an enhanced photodetector response.

**Fig. 4:**
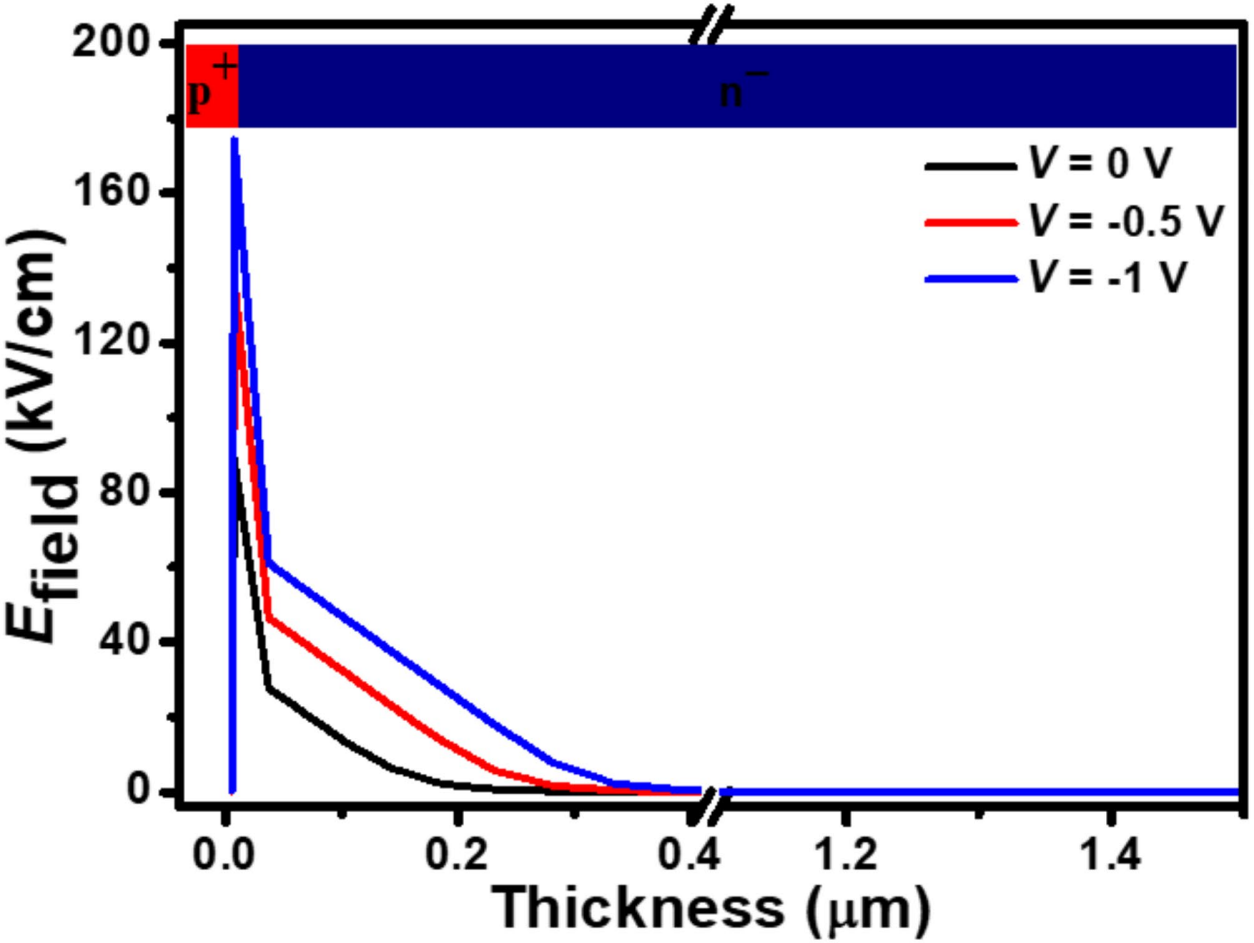
Simulated triangular-shaped built-in electric field profile for p^+^-FLG/n^–^-ZnO NWs heterojunction-based UV photodetector under various biasing conditions.

The alignment of the Fermi levels between p^+^-FLG and n^–^-ZnO NWs at equilibrium indicates a balance in chemical potential between the two materials. The electric field profile and energy bandgap diagram provide insights into the device’s ability to efficiently separate and collect photogenerated carriers, a key factor in high-performance photodetection.

Graphene layers have a broadband transparency of ~ 87%, allowing the incident light to penetrate in the NWs. The proposed photodetector shows susceptible optoelectronic properties through carrier generation or recombination during UV illuminations. Upon exposure to UV illumination, both the graphene and ZnO layers absorb incident energy, leading to the generation of electron-hole pairs within their respective bandgaps, as illustrated in Fig. 3(a). ZnO NWs can detect light at wavelengths beyond their normal optical bandgap of 350 nm through a process involving defects in their structure. Specifically, oxygen vacancies create energy states within the bandgap that allow the nanowires to absorb some UV light at 350 nm, resulting in the generation of photocarriers. The photogenerated carriers are efficiently separated and transported to the electrodes under the influence of $\:\overrightarrow{{E}_{\text{field}}}$ present at the interface of the p^+^-FLG and n^–^-ZnO NWs, thus improving the photoresponse. ZnO NWs also serve as a light-trapping medium, increasing the interaction length of photons and enhancing overall light absorption in the photodetector. Their vertically aligned structure maximizes the photogenerated carrier collection by reducing reflection losses and providing a high surface-to-volume ratio. Accordingly, the photogenerated electrons and holes are transported toward the n^–^-ZnO and p^+^-FLG, respectively, under applied reverse-biased conditions. This separation of photogenerated carriers leads to the generation of net photocurrent to the external circuit. The small bandgap in FLG creates the depletion layer of holes at the FLG side, whereas the large bandgap in the ZnO layers creates the depletion layer of electrons at the ZnO side. In Fig. 3(a), the M and K points represent specific locations in the Brillouin zone of graphene where incident photons are absorbed. The simulated energy bandgap diagram for the proposed photodetector under the self-biasing mode of operation is illustrated in Fig. 3(b). In Fig. 3(b), the *E*_VB_ (green line) break represents the abrupt band bending at the p⁺-FLG/n⁻-ZnO hetero-interface. This band bending arises due to the alignment of the Fermi levels of the two materials under equilibrium conditions, forming a strong built-in $\:\overrightarrow{{E}_{\text{field}}}$ at the heterojunction. The break signifies the potential barrier for separating photogenerated electron-hole pairs and enabling efficient charge transport. The triangular shape $\:\overrightarrow{{E}_{\text{field}}}$ profile across the heterojunction for the proposed photodetector under different reverse biasing conditions is demonstrated in Fig. 4. Such $\:\overrightarrow{{E}_{\text{field}}}$ is dominated over the heterojunction of p^+^-FLG and n^–^-ZnO and is enhanced by the external reverse bias. A maximum $\:\overrightarrow{{E}_{\text{field}}}$ of about 88.62, 136.82, and 174.14 kV/cm are found at 0, −0.5, and −1.0 V, respectively, for the proposed photodetector.

The performance of the photodetector is highly influenced by its biasing conditions. Under reverse bias conditions, an external voltage is applied in the opposite direction to the forward bias, which enhances the built-in electric field at the junction. This increased electric field improves the separation and collection of photogenerated electron-hole pairs, leading to a higher photocurrent. The resulting electric field profile directly influences the device’s performance, as seen in the enhanced values of the electric field with increasing reverse bias (Fig. 4). Understanding the influence of biasing on the device’s performance is essential for optimizing its optoelectronic properties.

The proposed photodetector operates in two distinct modes: self-biasing and photoconductive. In self-bias mode, the device operates without external power, relying solely on the internal electric field generated at the heterojunction. This mode is advantageous for low-power applications, as it minimizes power consumption and circuit complexity. The photodetector demonstrates a significant photovoltaic response under zero bias conditions, indicative of its self-biasing operation.

In photoconductive mode, an external reverse bias enhances the device’s performance. This mode increases the electric field at the heterojunction, resulting in more efficient separation and collection of photogenerated carriers. The application of reverse bias improves the photodetector’s photocurrent responsivity, quantum efficiency, and overall speed, making it suitable for high-performance applications. The observed variation in photocurrent with different reverse bias voltages further illustrates the device’s responsiveness to external biasing. Understanding these biasing effects is crucial for optimizing the photodetector’s optoelectronic properties and tailoring its performance to specific applications, allowing for a balance between power efficiency and high performance as needed.

Since the graphene layers have atom-thin thickness, resulting in the creation of the depletion region width either almost very thin (≈ 2.81 × 10^–13^ m) or fully depleted, hence, is ignored in this work. NWs-based photodetector, mechanism models such as SRH and Auger are used. Analytically speaking, the net *J*_dark_ that depends on the applied reverse bias voltage (*V*) and temperature *T* for the proposed ZnO NWs-based photodetector comprises the diffusion current density (*J*_DIFF_) and generation-recombination (G-R) of charge carriers induced drift current density (*J*_GR_) at the heterojunction^40,57^ i.e.

11 $$\:{J}_{\text{d}\text{a}\text{r}\text{k}}\left(V,\text{T}\right)=\:{J}_{\text{D}\text{I}\text{F}\text{F}}+{J}_{\text{G}\text{R}}$$

where

12 $$\:{J}_{\text{DIFF}}=\hspace{0.33em}\left[{\left({J}_{{\text{n}}^{-}}\right)}_{{\text{p}}^{\text{+}}}+{\left({J}_{{\text{p}}^{\text{+}}}\right)}_{{\text{n}}^{-}}\hspace{0.33em}\right]\hspace{0.33em}\left({e}^{\left(qV/{k}_{B}T\right)}-1\right)$$

here $\:{\left({J}_{{\text{n}}^{-}}\right)}_{{\text{p}}^{\text{+}}}$ and $\:{\left({J}_{{\text{p}}^{\text{+}}}\right)}_{{\text{n}}^{-}}$ signify the diffusion component of current density for electrons and holes in p^+^-FLG and n^–^-ZnO regions, respectively, and are modeled as^40,57^

13 $$\:{\left({J}_{{\text{n}}^{-}}\right)}_{{\text{p}}^{\text{+}}}=\frac{q{n}_{\text{i,}\text{p}}^{2}}{{N}_{\text{A}}}\sqrt{\frac{{\mu\:}_{\text{n}}{k}_{\text{B}}T}{q{\tau\:}_{\text{n}}}}\frac{{S}_{\text{n}}{L}_{\text{n}}+{D}_{\text{n}}\text{tanh}\left(\frac{{{t}}_{\text{p}}-{x}_{\text{p}}}{{L}_{\text{n}}}\right)}{{D}_{\text{n}}+{S}_{\text{p}}{L}_{p}\text{tanh}\left(\frac{{{t}}_{\text{p}}-{x}_{\text{p}}}{{L}_{\text{n}}}\right)}$$

14 $$\:{\left({J}_{{\text{p}}^{\text{+}}}\right)}_{{\text{n}}^{-}}=\frac{q{n}_{\text{i,}\text{n}}^{2}}{{N}_{\text{D}}}\sqrt{\frac{{\mu\:}_{\text{p}}{k}_{B}T}{q{\tau\:}_{\text{p}}}}\frac{{S}_{\text{p}}{L}_{\text{p}}+{D}_{\text{p}}\text{tanh}\left(\frac{d-{x}_{\text{n}}}{{L}_{\text{p}}}\right)}{{D}_{\text{p}}+{S}_{\text{p}}{L}_{\text{p}}\text{tanh}\left(\frac{d-{x}_{\text{n}}}{{L}_{\text{p}}}\right)}$$

In the above mathematical expressions, $\:\:{n}_{\text{i},\text{p}}$and $\:{n}_{\text{i},\text{n}}$ are the intrinsic carrier concentrations in p^+^-FLG and n^–^-ZnO layers, respectively; *N*_A_ and *N*_D_ represent the acceptor and donor ions concentration, respectively. The surface recombination velocities of electrons and holes at the p^+^-FLG/n^–^-ZnO heterojunction are denoted by *S*_n_ and *S*_p_, respectively. $\:{L}_{\text{n}}$ and $\:{L}_{\text{p}}$ are the diffusion lengths of electrons and holes, respectively. $\:{{t}}_{\text{p}}$ and *d* represents the thickness of p^+^-FLG and n^–^-ZnO layers, respectively. The width of the depletion region in the p^+^-FLG and n^–^-ZnO layers is denoted by *x*_p_ and *x*_n_, respectively. When the UV light is incident with an incident power density *P*_in_ of 1 W/cm^2^, the charge carriers are generated, hence, photocurrent flows. The total photocurrent density (*J*_light_) through the photodetector is given by^3^.

15 $$\:{J}_{\text{light}}={J}_{\text{dark}}-\frac{Q{E}_{\text{ext}}q{\lambda\:}_{c}{P}_{\text{in}}}{hc}={J}_{\text{dark}}-\frac{Q{E}_{\text{ext}}{\lambda\:}_{c}{P}_{\text{in}}}{1.24}$$

where *QE*_ext_, λ_c_, *h*, and *c* represent the external quantum efficiency, center or cut-off wavelength, Planck’s constant, and speed of light, respectively.

**Fig. 5:**
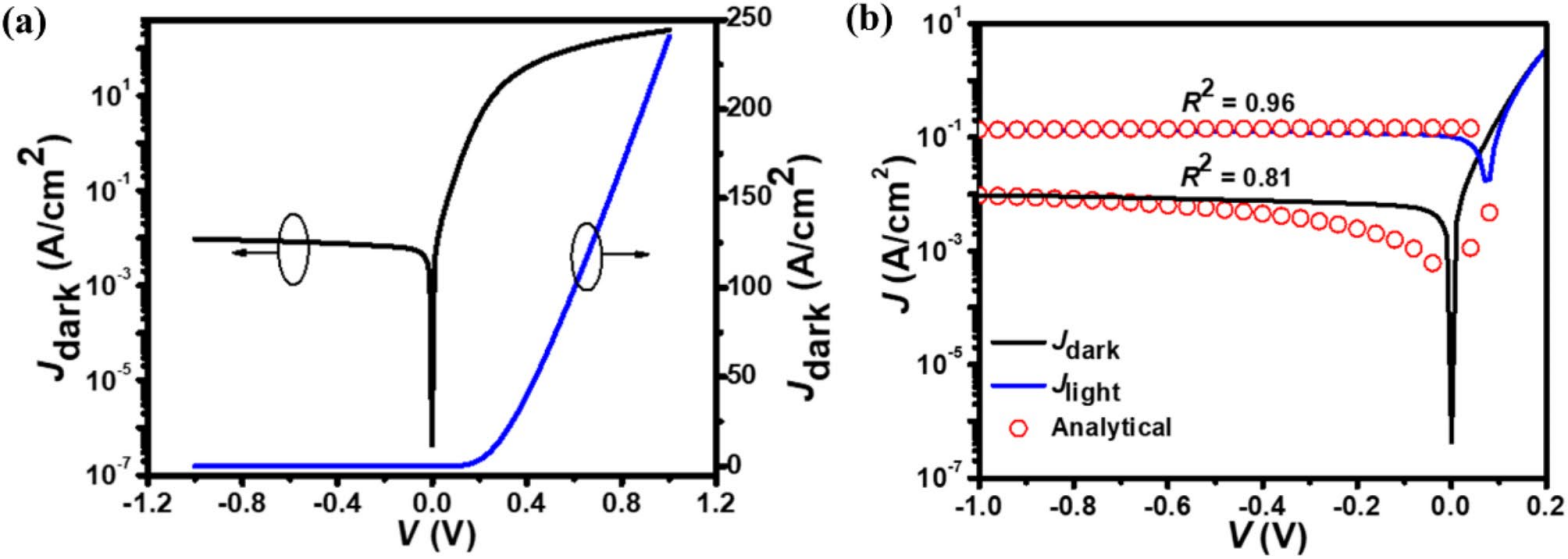
**(a)** The simulated *J*_dark_-*V* characteristic of the p^+^-FLG/n^–^-ZnO NWs photodetector in logarithmic and linear scale at 300 K, demonstrating the current rectification behavior, indicative of the diode-like operation of the device under dark conditions. **(b)** The simulated *J*-*V* characteristic of the p^+^-FLG/n^–^-ZnO NWs photodetector under the dark and UV illumination at 350 nm wavelength with an intensity of 1 W/cm^2^, illustrating both photovoltaic and photoconductive modes of operation. The close match between the experimental data (solid curves) and analytical modeling (open symbols) highlights the accuracy of the theoretical model, as evidenced by *R*^2^ values of 0.81 and 0.96 under dark and illuminated conditions, respectively.

The room temperature current density-voltage (*J*-*V*) characteristics of the proposed photodetector under different biasing conditions and at different wavelengths are shown in Fig. 5. The *J*-*V* characteristics at 300 K demonstrate a significant increase in current densities with reverse bias, highlighting the importance of biasing in tuning the device’s performance.

Figure 5(a) illustrates the rectifying behavior of the p^+^-FLG/n^–^-ZnO NWs-based heterojunction, which is crucial for efficient charge separation. The *J*_dark_-*V* characteristic curve of the photodetector shown in both logarithmic and linear scales (Fig. 5(a)), results in a high current rectification ratio of 2.5 × 10^4^ at ±1 V, which is better than multilayer WSe_2_/ZnO photodetector^2^. This high rectification ratio indicates a well-formed depletion region at the interface, enhancing the device’s ability to separate photogenerated carriers. This rectification behavior confirms the effective formation of a diode-like junction at the p^+^-FLG/n^–^-ZnO, which is crucial for the efficient separation of photo-generated carriers.

The simulated *J*-*V* characteristics of the photodetector under both dark and 350 nm wavelength (λ) illumination in logarithmic scale are shown in Fig. 5(b). As can be seen, the current density depends on the applied voltage and increases significantly with voltage under illumination. This behavior indicates the photodetector’s strong photoresponse, driven by the efficient carrier generation and separation at the heterojunction interface. The relatively smaller reverse current density compared to the forward current density, indicates the formation of a barrier at the p^+^-FLG/n^–^-ZnO hetero-interface, which enhances the photodetector’s selectivity for UV light.

The photodetector exhibits a *J*_dark_ value of 0.4 µA/cm^2^ and 8 mA/cm^2^ under the self-biasing and photoconductive (i.e., at −0.5 V) modes, respectively. On the other hand, the *J*_light_ values of 0.1 and 0.13 A/cm^2^ under self-biasing and −0.5 V are found at the center wavelength of 350 nm. The estimated value of *J*_light_ at 0 V clearly describes the photovoltaic behavior of the photodetector. The same kind of self-biasing behavior for graphene-based photodetectors is also noticed in^34,81,82^. Such an improvement in the *J*_light_ from the *J*_dark_ is due to the efficient generation of carriers under the UV exposure at the p^+^-FLG/n^–^-ZnO NWs heterojunction, with the carriers being drifted by the $\:\overrightarrow{{E}_{\text{field}}}$ present at the heterojunction. As illustrated in Fig. 5(b), the simulated *J*-*V* characteristic curves closely match the values obtained from analytical modeling, with a correlation *R*^2^ value of 0.81 and 0.96 under dark and illumination conditions, respectively. The close match confirms the robustness of the analytical model. For the analytical modeling, the considered values for the *S*_n_ and *S*_p_ are 10^5^ and 100 cm/s, respectively. Under self-biasing mode, the proposed photodetector’s *J*_light_*/J*_dark_ ratio is 2.5 × 10^5^. The measured *J*_dark_, *J*_light_, and *J*_light_/*J*_dark_ values are found to be better than the values reported in earlier works^6,81,83–87^.

**Fig. 6:**
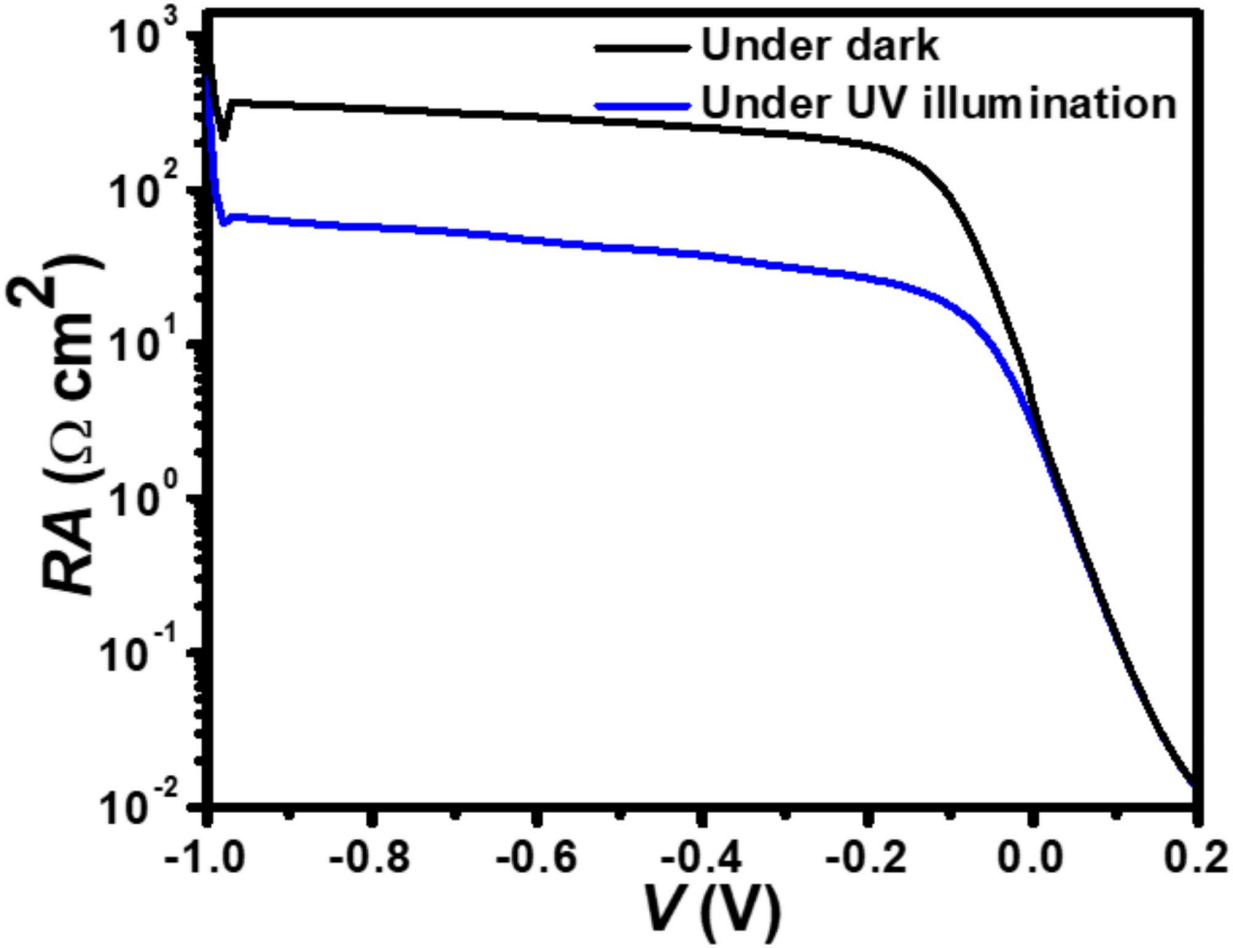
The simulated *RA*-*V* characteristic of the p^+^-FLG/n^–^-ZnO NWs photodetector under the dark and UV illumination at 350 nm wavelength with an intensity of 1 W/cm^2^.

Figure 6 shows the variation of total resistance (*R*) area (*A*) product $\:\left({RA}={\left(\frac{\text{d}{J}}{\text{d}{V}}\right)}^{-1}\right)$ with reverse bias voltage for both in the absence and existence of UV exposure (350 nm wavelength) conditions. The value of *RA* is high in the absence of UV illuminations due to the lower conductivity of the proposed photodetector. However, under UV exposure, the *RA* value decreases with the applied bias voltage due to the generation of increased free charge carriers. This decrease in *RA* product under illumination reflects the photoconductivity effect, where UV-generated carriers reduce the overall resistance, thereby enhancing the device’s sensitivity to light. At 0 V, the *RA* value for the proposed photodetector under dark and illumination conditions is found to be 4.02 and 2.97 Ωcm^2^, respectively. On the other hand, at −0.5 V, the *RA* value under dark and illumination conditions is found to be 275.21 and 41.89 Ωcm^2^, respectively, indicating a substantial decrease in resistance with UV exposure, which enhances the photodetector’s responsivity.

**Fig. 7:**
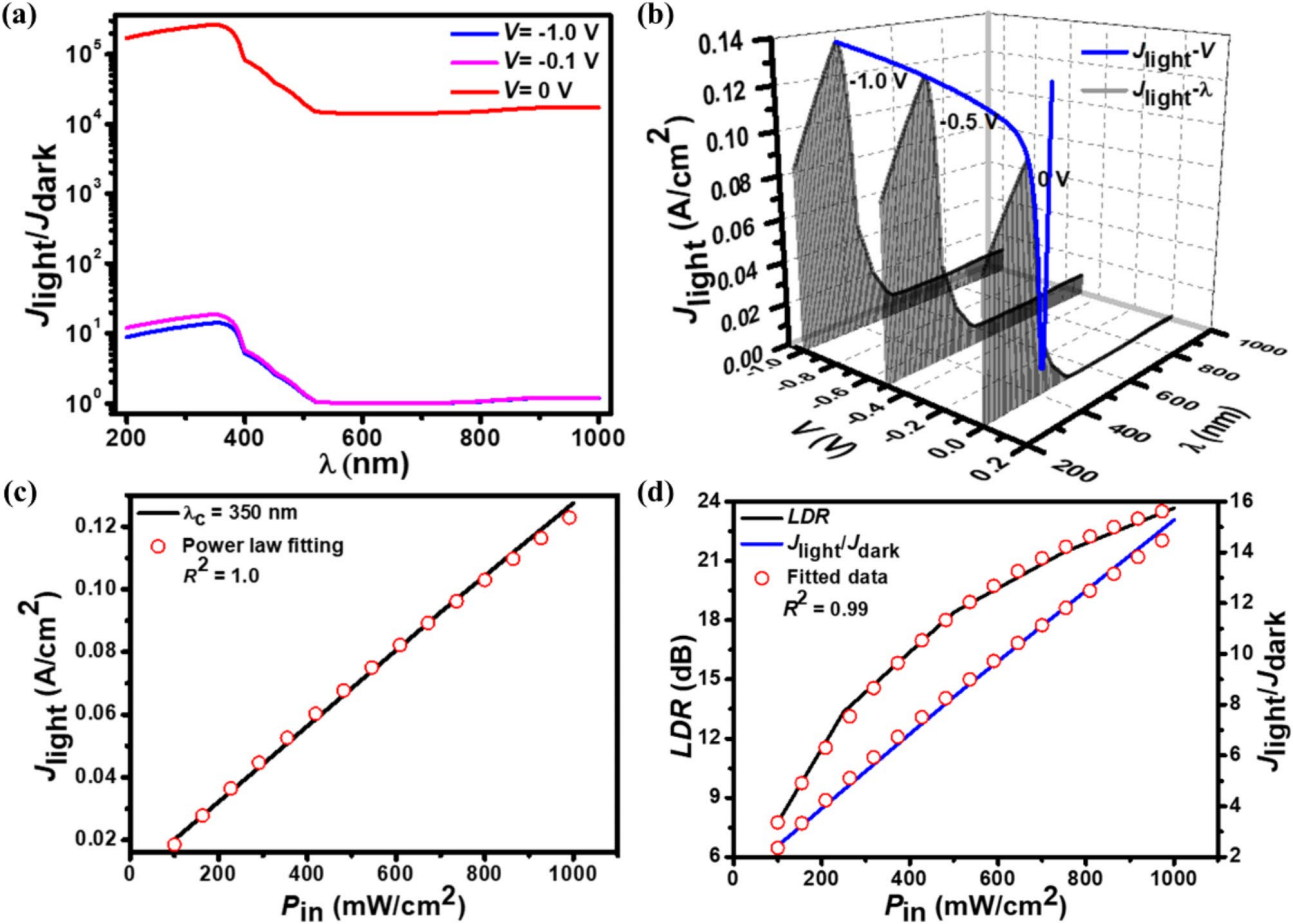
**(a)** The simulated plot of *J*_light_/*J*_dark_ ratio with applied wavelength at different reverse biased voltages. **(b)** 3D simulated plot of *J*_light_ as a function of applied voltage and wavelength at 1 W/cm^2^ and 300 K. The *J*_light_-*λ* curve plotted at 0, −0.5, and −1.0 V shows maximum *J*_light_ at the center wavelength of 350 nm wavelength. **(c)** Simulated *J*_light_-*P*_in_ characteristics at −0.5 V under different wavelengths and 300 K. *J*_light_-*P*_in_ curves has been fitted with a simple power-law (open symbols) having a correlation *R*^2^ value of 1.0. **(d)** The variation of *LDR* and *J*_light_/*J*_dark_ ratio with *P*_in_ at −0.5 V and 350 nm center wavelength. The *LDR*-*P*_in_ and *J*_light_/*J*_dark_-*P*_in_ have been fitted resulting in a correlation *R*^2^ value of 0.99.

Figure 7(a) depicts the *J*_light_/*J*_dark_ ratio with incident wavelength at 0, −0.1, and −1.0 V. It is noted that with the increase in wavelength from 200 to 1000 nm, the *J*_light_/*J*_dark_ ratio is first increased and then begins decreasing. The *J*_light_/*J*_dark_-*λ* curve shows a peak value at 350 nm due to the generation of a maximum value of *J*_light_ at this wavelength. It is further noticed that with the increase in reverse biased voltage, the *J*_light_/*J*_dark_ ratio decreases. This decrease in *J*_light_/*J*_dark_ ratio is attributed to the increase in *J*_dark_ value with the reverse biased voltage. Figure 7(b) shows the 3D plot for *J*_light_ as a function of applied reverse bias voltage and *λ* for the proposed NWs-based heterojunction photodetector with a *P*_in_ of 1 W/cm^2^ at 300 K. The spectral response (*J*_light_-*λ*) plotted at 0, − 0.5, and − 1.0 V shows the peak value of *J*_light_ at 350 nm wavelength. This peak response at 350 nm demonstrates the device’s spectral sensitivity and its optimization for UV detection, confirming the wavelength-selective behavior with the strongest photoresponse in the UV region. Since the incident optical energy absorbed by any photosensitive material is limited by its energy bandgap, the *J*_light_-*λ* curve (Fig. 7(b)) shows a maximum value before reaching its center wavelength *λ*_c_. Moreover, the *J*_light_ decreases due to low absorbance and high optical transmittance of light, signifying that the light is barely absorbed by the material after a particular wavelength. A similar photoresponse profile for graphene/ZnO NWs-based heterojunction photodetector is described with maxima around this wavelength in^52^.

The *J*_light_ variation due to different values of *P*_in_ at a bias of −0.5 V under 350 nm wavelength is depicted in Fig. 7(c). The *J*_light_ of the proposed photodetector is observed to exhibit perfect linearity with increasing illumination intensities. Specifically, as *P*_in_ increases from 100 to 1000 mW/cm^2^, *J*_light_ rises linearly from 0.02 to 0.13 A/cm^2^. The linear response of *J*_light_ with *P*_in_ further results in an almost linear variation of photocurrent density-to-dark current density ratio, photocurrent responsivity, and detectivity with incident *P*_in_. The quantitative study of the relationship between *J*_light_ and *P*_in_ shows that the *J*_light_ value can be fitted with *P*_in_ by a simple power-law $\:{J}_{\text{light}}=a{P}_{\text{in}}^{\alpha\:}$^3^, where *a* is a constant, and *α* corresponds to the fitting parameter associated with the recombination process of the photogenerated charge carriers. After fitting the equation to the simulated results gives $\:{J}_{\text{light}}=0.13{P}_{\text{in}}^{0.99}$ with a correlation *R*^2^ value of 1.0. The linear response in *J*_light_-*P*_in_ curve indicates that the recombination loss is insignificant^40^. The *J*_light_ value for the proposed photodetector is better than that of earlier stated graphene/ZnO and graphene/Si heterojunction-based photodetectors^34,38,81^. The reason for such high performance is due to the enhanced absorption by graphene layers and a huge electric field at the hetero-interface of p^+^-FLG and n^–^-ZnO NWs, which rapidly drifts the photoexcited carriers to the external electrodes, contributing to *J*_light_.

The linear dynamic range $\:\left({LDR}\left(\text{dB}\right)\text{=20log}\left({J}_{\text{light}}/{J}_{\text{dark}}\right)\right)$ is a vital indicator for photodetector performance, representing the range over which the device maintains a linear response. For the proposed p⁺-FLG/n⁻-ZnO NW-based UV photodetector, the *LDR* reaches up to 24.2 dB, enabling accurate photodetection across a broad range of UV intensities. Figure 7(d) shows the variation of *LDR* and *J*_light_/*J*_dark_ ratio with *P*_in_ at a bias of −0.5 V. The *LDR* value rises exponentially from 8 to 24.2 dB with *P*_in_ varied from 100 to 1000 mW/cm^2^. A large value of the *LDR* is necessary for real-world applications to sense both weak and strong light. Quantitatively, the *LDR* fits an exponential relation with *P*_in_ by the expression

16 $$\:{{LDR} \:=\: {A}}_{0}+{{A}}_{1}{e}^{\left(\frac{{P}_{in}}{{t}_{1}}\right)}.$$

where *A*_0_, *A*_1_, and *t*_1_ are the fitting parameters. After fitting the equation to the simulated results, it gives *A*_0_ = 26.72, *A*_1_ = − 23.26, and *t*_1_ = − 491.03 with *R*^2^ = 0.99. On the other hand, the *J*_light_/*J*_dark_ ratio varies from 2.4 to 16.25 linearly with *P*_in_ in a simple power-law manner by the expression $\:{J}_{\text{light}}/{J}_{\text{dark}}=\text{0.06}{P}_{\text{in}}^{0.8}$ having *R*^2^ = 0.99.

**Fig. 8:**
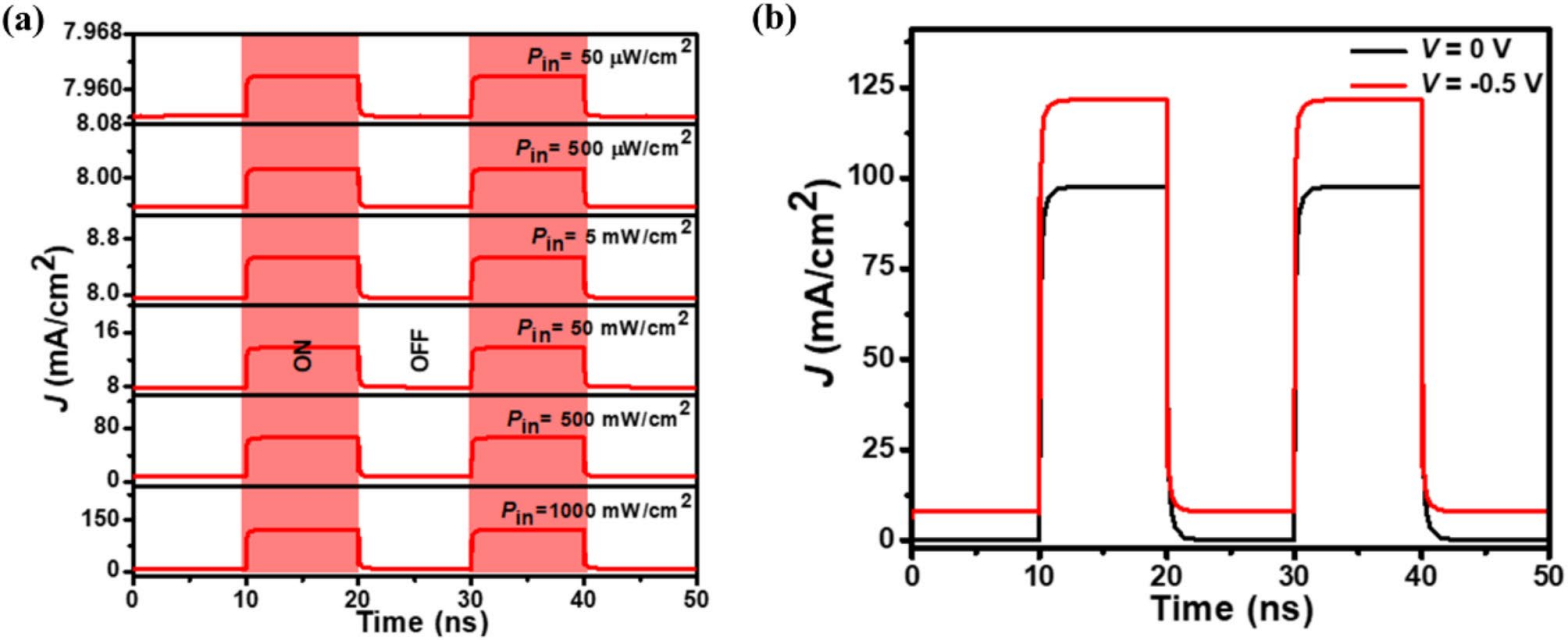
The simulated photoswitching characteristics of the p^+^-FLG/n^–^-ZnO NWs photodetector. **(a)** *J*-Time characteristics under different *P*_in_ values at 350 nm and −0.5 V. **(b)** *J*-Time characteristics at 350 nm wavelength under 0 V and −0.5 V with 1 W/cm^2^ illumination intensity.

To study the photodetection speed, i.e., the maximum bit rate that the photodetector can detect, the time-dependent photoswitching characteristics (*J*-Time) under the UV light illumination are explored. Figure 8(a) depicts the *J*-Time characteristics of the proposed photodetector under different UV illumination intensities with λ_c_ = 350 nm at −0.5 V bias. At the instant of UV exposure with a different *P*_in_, the *J*_light_ increases, while in the absence of UV exposure, the *J*_light_ decreases abruptly to an initial *J*_dark_ value of 8 mA/cm^2^. To further evaluate the self-biasing and photoconductive photodetection performance of the photodetector, the *J*-Time characteristics are measured at an excitation wavelength of 350 nm under 0 and −0.5 V (Fig. 8(b)). As shown, the increase in reverse bias voltage results in an increased *J*_light_ value. It is further noted that the *J*_light_ decreases with excitation wavelength, showing the highest value at 350 nm of 97.75 and 121.67 mA/cm^2^ under self-biasing and photoconductive modes, respectively. This is due to the low absorbance and high optical transmittance of light, signifying that the light is barely absorbed by the n^–^-ZnO NWs after a specific wavelength^88^.

Both the rise time (*τ*_r_) and fall time (*τ*_f_) are found to be 0.26 ns at 350 nm wavelength under self-biasing mode. On the other hand, at −0.5 V bias, the *τ*_r_ and *τ*_f_ are both found to be 0.16 ns at 350 nm wavelength. It is noted that sharp and rapid photoswitching is obtained for the proposed photodetector and is necessary for the high-performance photodetector. The photoswitching time will increase with the wavelength and show a faster rise and fall time at 350 nm. It is also observed that the *τ*_r_ (*τ*_f_) varies from 0.16 to 0.17 ns (0.15 to 0.17 ns) when the *P*_in_ varies from 100 to 1000 mW/cm^2^ at 350 nm wavelength (Fig. S2). The measured *τ*_r_ and *τ*_f_ are significantly better than the previously reported works^35,38,81,89–95^. The photodetector’s enhanced response speed can be attributed to two main factors: the higher carrier mobility in the graphene layers and the absence of interface defects in the simulation. While graphene is renowned for its extraordinary electron mobility; real-world performance may be affected by imperfections at the FLG/ZnO interface. These imperfections, which can arise during fabrication, were not fully incorporated into the simulation. Consequently, while the simulation provides insight into the potential performance of the idealized device, the response speed results should be interpreted with caution when compared to experimental data from other detectors. To align the simulation results more closely with actual device performance, future experimental studies should account for these interface imperfections and potential carrier traps, allowing for a more realistic evaluation of response speed in practical applications. The 3-dB cut-off frequency $\:\left({f}_{\text{3-dB}}\text{=}\frac{\text{0.34}}{{τ}_{\text{r}}}\right)$, where the photocurrent drops to 70% of its peak value^40^ and is found to be 1.31 and 2.13 GHz under 0 and −0.5 V, respectively at 350 nm.

The optical characterization of the proposed p^+^-FLG/n^–^-ZnO NWs-based heterojunction photodetector is studied in terms of external photocurrent responsivity $\:\left({R}_{\text{i}}^{\text{ext}}\right)$, external quantum efficiency (*QE*_ext_), detectivity (D^*^), and noise equivalent power (*NEP*). The $\:{R}_{\text{i}}^{\text{ext}}$, *QE*_ext_, D^*^, and *NEP* values with respect to wavelength are calculated by using the expressions^9,40,96,97^

17 $$\:{R}_{\text{i}}^{\text{ext}}\text{=}\frac{{J}_{\text{light}}}{{P}_{\text{in}}}\:\:\:\:\text{A/W}$$

18 $$\:{QE}_{\text{ext}}\text{=}\frac{{hc}{R}_{\text{i}}^{\text{ext}}}{{q \lambda}} \times 100 \text{=}\frac{1.24{R}_{\text{i}}^{\text{e}\text{x}\text{t}}}{{\uplambda\:}}\times100\:\:\:\:\%$$

19 $$\:{D}^{\text{*}}=\frac{{R}_{\text{i}}^{\text{e}\text{x}\text{t}}}{2}\sqrt{\frac{\left({R}_{0}A\right)}{{k}_{\text{B}}T}}\:\:\text{Jones}$$

20 $$\:{NEP}\text{=}\frac{\sqrt{\triangle{fA}}}{{{D}}^{\text{*}}}\:\:\:\:\:\text{W/}{\text{Hz}}^{\text{1/2}}$$

where (*R*_0_*A*) is the resistance area product under the self-biasing mode, and Δ*f* is the bandwidth. In this work, the *NEP* is calculated at Δ*f* = 1 Hz.

Quantitatively, the *QE*_ext_ of the proposed photodetector comprises three regions, the neutral p^+^-, the neutral n^–^-, and the depletion regions^40,57^ i.e.

21 $$\:{QE}_{\text{ext}}=({QE}_{\text{ext}}{)}_{{\text{p}}^{+}}+({QE}_{\text{ext}}{)}_{{\text{n}}^{-}}+({QE}_{\text{ext}}{)}_{\text{depletion}}$$

where

$$(QE_{\rm ext})_{p} {+} = \frac{(1-{R}_{p}) \alpha_{p}{L}_{n}}{\alpha _{p}^{2}{L}_{n}^{2}-1}$$

21a $$\:{e}^{-\left({\alpha}_{\text{p}}{t}_{\text{p}}+{\alpha}_{\text{n}}{x}_{\text{n}}\right)}\times\:\left[\frac{\left(\frac{{S}_{\text{n}}{L}_{\text{n}}}{{D}_{\text{n}}}-{\alpha}_{\text{p}}{L}_{\text{n}}\right){e}^{-{\alpha}_{\text{n}}\text{(}{d}-{x}_{\text{n}})}-\left\{\frac{{S}_{\text{n}}{L}_{\text{n}}}{{D}_{\text{n}}}\text{cosh}\left(\frac{{d}-{x}_{\text{n}}}{{L}_{\text{n}}}\right)+\text{sinh}\left(\frac{{d}-{x}_{\text{n}}}{{L}_{\text{n}}}\right)\right\}}{\text{cosh}\left(\frac{{d}-{x}_{\text{n}}}{{L}_{\text{n}}}\right)+\frac{{S}_{\text{n}}{L}_{\text{n}}}{{D}_{\text{n}}}\text{sinh}\left(\frac{{d}-{x}_{\text{n}}}{{L}_{\text{n}}}\right)}+{\alpha}_{\text{p}}{L}_{\text{n}}\right]\:$$

21b $$\:({QE}_{\text{ext}}{)}_{{\text{n}}^{-}}=\frac{(\text{1}-{R}_{\text{p}})(1-{R}_{\text{n}}){\alpha}_{\text{n}}{L}_{\text{p}}}{{\alpha}_{\text{n}}^{\text{2}}{L}_{\text{p}}^{\text{2}}-\text{1}}\times\:\left[\frac{\left({\alpha}_{\text{n}}{L}_{\text{p}}+\frac{{S}_{\text{p}}{L}_{\text{p}}}{{D}_{\text{p}}}\right)-{e}^{-{\alpha}_{\text{n}}{x}_{\text{p}}}\left\{\frac{{S}_{\text{p}}{L}_{\text{p}}}{{D}_{\text{p}}}\text{cosh}\left(\frac{{x}_{\text{p}}}{{L}_{\text{p}}}\right)+\text{sinh}\left(\frac{{x}_{\text{p}}}{{L}_{\text{p}}}\right)\right\}}{\text{cosh}\left(\frac{{x}_{\text{p}}}{{L}_{\text{p}}}\right)+\frac{{S}_{\text{p}}{L}_{\text{p}}}{{D}_{\text{p}}}\text{sinh}\left(\frac{{x}_{\text{p}}}{{L}_{\text{p}}}\right)}-{\text{}\alpha}_{\text{n}}{L}_{\text{p}}{e}^{-{\alpha}_{\text{n}}{x}_{\text{p}}}\right]$$

and

21c $$\:({QE}_{\text{ext}}{)}_{\text{depletion}}=(\text{1}-{R}_{\text{p}}\left)\right(1-{R}_{\text{n}})\times\:\left[{e}^{-{\alpha}_{\text{p}}{x}_{\text{p}}}-{\text{e}}^{-{\alpha}_{\text{n}}({t}_{\text{p}}+{x}_{\text{n}})}\right]$$

where *R*_p_ and *R*_n_ represent the Fresnel’s reflection coefficients^58^ at the entrance and p^+^-FLG/n^–^-ZnO NWs hetero-interface, respectively.

**Fig. 9:**
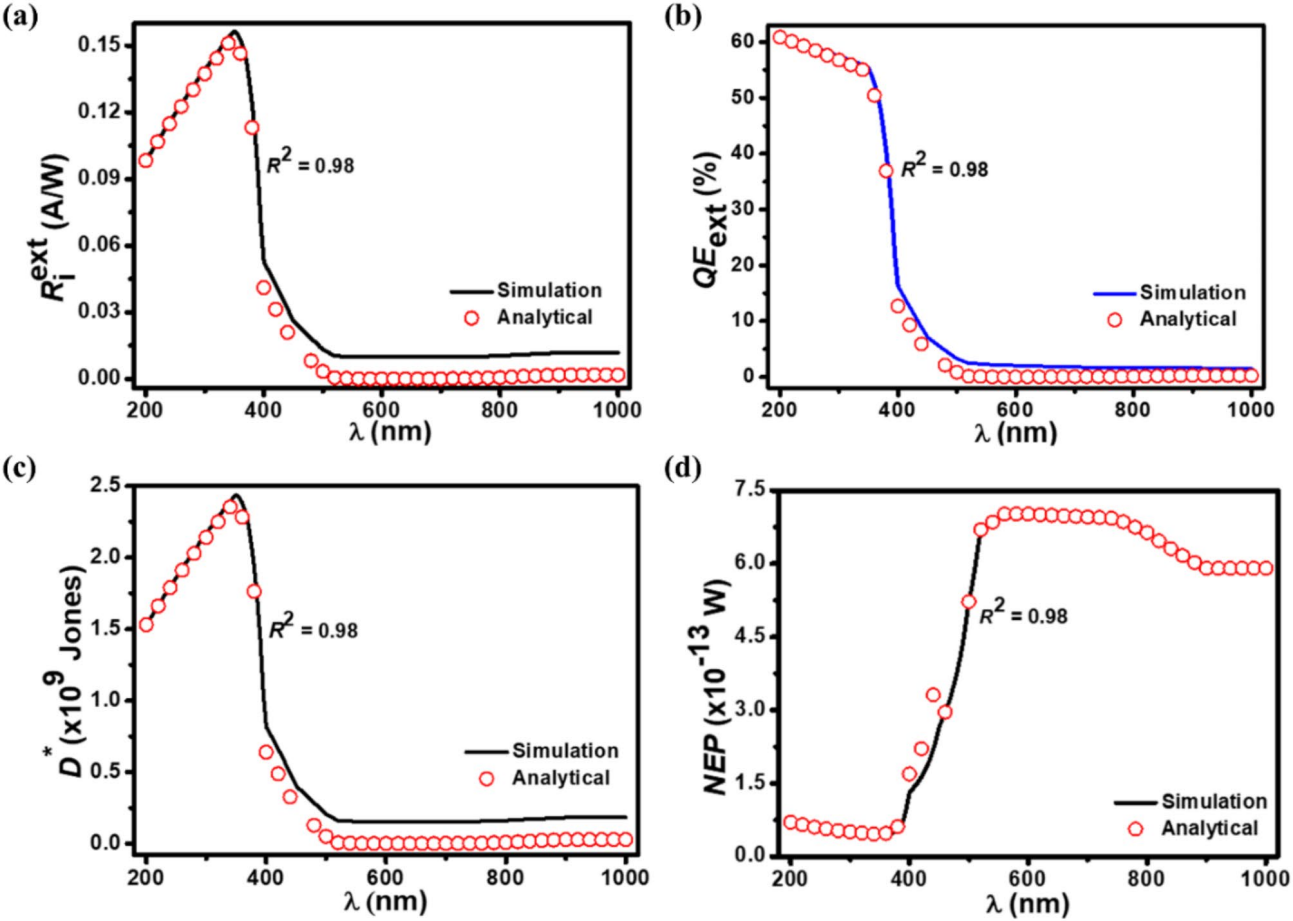
The simulated optical characteristics of the p^+^-FLG/n^–^-ZnO NWs photodetector under −0.5 V with 1 W/cm^2^ illumination intensity at 350 nm. **(a)** $\:{R}_{\text{i}}^{\text{ext}}$-λ characteristics. **(b)** *QE*_ext_-λ characteristics. **(c)** D^*^-λ characteristics. **(d)** *NEP*-λ characteristics. The simulated optical characteristic curves (solid curves) closely match the values obtained from analytical modeling (open symbols) having a correlation *R*^2^ value of 0.98.

Figure 9(a)-9(d) show the dependence of $\:{R}_{\text{i}}^{\text{ext}},$ *QE*_ext_, D^*^, and *NEP* for the proposed NWs-based photodetector with the excitation wavelength under 1 W/cm^2^, −0.5 V, and 300 K. It is clear that the simulated results of optical characteristic parameters of the proposed photodetector perfectly match the analytical expressions with a correlation *R*^2^ value of 0.98. It is seen from the $\:{R}_{\text{i}}^{\text{ext}}$-λ and D^*^-λ characteristic curves of the proposed photodetector that both $\:{R}_{\text{i}}^{\text{ext}}$ and D^*^ initially increases with the wavelength from 200 to 350 nm to a point and after that start to decrease with wavelength. The $\:{R}_{\text{i}}^{\text{ext}}$ and D^*^ reach their peak value of 0.2 A/W and 2.4 × 10^9^ Jones, respectively, at 350 nm wavelength. The reason for such a decrease after this center wavelength is the low absorbance and high optical transmittance of incident light by the materials. This indicates that the light is hardly absorbed by the n^–^-ZnO NWs after a center wavelength. The *QE*_ext_ and *NEP* of the proposed photodetector are estimated to be about 56% and 4.4 × 10^–14^ W, respectively, at 350 nm wavelength. The maximum value of *QE*_ext_ of about 61% is found for the proposed photodetector. The minimum value of *NEP* is beneficial to realize sensitive photodetection and improved performance of the photodetector. Such a high performance is credited to the higher mobility of graphene and utilization of more graphene layers, which further enhances the optical absorbance in the p^+^-FLG/n^–^-ZnO NWs heterojunction. The existence of strong electric field (~ 136.82 kV/cm) at the p^+^-FLG/n^–^-ZnO NWs heterojunction, which efficiently separates the photoexcited carriers is another reason for the enhanced performance.

The experimental error in this work primarily arises from uncertainties in material properties, fabrication processes, and measurement conditions. Slight variations in key parameters such as doping concentrations, layer thicknesses, and NW dimensions can compromise the uniformity of the photodetector structure, potentially impacting overall performance. Environmental factors like temperature fluctuations may introduce additional variability. To mitigate these errors, simulation tools were employed with precise boundary conditions, and results were validated using analytical modeling. The close agreement between simulated and theoretical results (*R*² values exceeding 0.95) ensures errors remain within an acceptable range. The optimized geometry and configurations reflect realistic fabrication tolerances, enhancing reliability. The estimated margin of error is within ±5%, and future experimental studies will include statistical analyses of fabricated devices to quantify and reduce these errors.

**Fig. 10:**
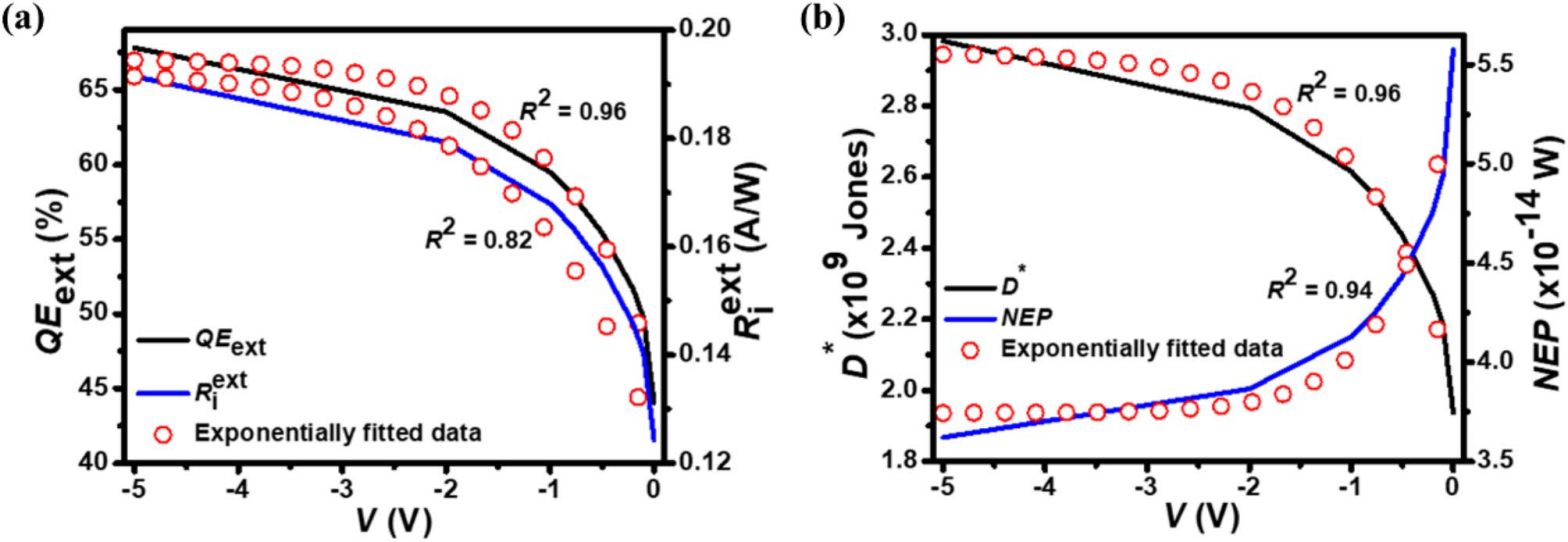
The effect of applied reverse bias voltage on the optical characteristics of p^+^-FLG/n^–^-ZnO NWs photodetector at 350 nm with an illumination of 1 W/cm^2^. (a) *QE*_ext_-*V* and $\:{R}_{\text{i}}^{\text{ext}}$-*V* characteristics. (b) D^*^-*V* and *NEP*-*V*. The simulated optical characteristic curves (solid curves) have been fitted exponentially (open symbols) having a correlation *R*^2^ value in the range of 0.82–0.96.

The effect of applied reverse bias voltage on the optical characteristics of the photodetector at 350 nm with an illumination of 1 W/cm^2^ is shown in Fig. 10(a) and 10(b). The $\:{R}_{\text{i}}^{\text{ext}}$, *QE*_ext_, and D^*^ increase exponentially from 0.12 to 0.2 A/W, 44.1 to 68%, and 1.9 × 10^9^ to 3 × 10^9^ Jones, respectively, with the applied bias voltages *V* varying from 0 to −5.0 V at fixed illumination intensity. As stated above, the increase in reverse bias voltage will enhance the photocurrent density. Alternatively, with the increase in external bias, the electric field across the photodetector is also enhanced. Consequently, it increases the charge collection efficiency on the electrodes by minimizing the electron-hole pair recombination. Therefore, $\:{R}_{\text{i}}^{\text{ext}}$ increases with the applied reverse bias voltage resulting in increased values of *QE*_ext_ and D^*^. Quantitatively the $\:{R}_{\text{i}}^{\text{ext}}$, *QE*_ext_, and D^*^are fitted according to the expression $\:{A}_{2}+{A}_{3}{e}^{\left(\frac{V}{{t}_{2}}\right)}\:$with *R*^2^ in the range of 0.82–0.96. On the other hand, the *NEP* decreases exponentially from 3.6 × 10^–14^ to 5.6 × 10^–14^ W with applied reverse bias voltage varying from −5.0 to 0 V according to the expression $\:{A}_{2}+{A}_{3}{e}^{\left(-\frac{V}{{t}_{2}}\right)}$ with *R*^2^ = 0.94. Here *A*_2_, *A*_3_, and *t*_2_ are the fitted parameters. For $\:{R}_{\text{i}}^{\text{ext}}$-*V* curve, the values of *A*_2_ = 0.19, *A*_3_ = − 0.07, and *t*_2_ = 1.24 are found. For *QE*_ext_-*V* curve, *A*_2_ = 67.08, *A*_3_ = − 20.85, and *t*_2_ = 0.93. The D^*^-*V* curve results in *A*_2_ = 2.95 × 10^9^, *A*_3_ = − 9.17 × 10^8^, and *t*_2_ = 0.93. The *NEP*-*V* curve gives *A*_2_ = 3.74 × 10^–14^, *A*_3_ = 1.62 × 10^–14^, and *t*_2_ = − 0.59. The improvement in $\:{R}_{\text{i}}^{\text{ext}}$, *QE*_ext_, D^*^, and *NEP* with the applied reverse bias voltage are attributed to the influence of photogenerated charge carriers, increased possibility of exciton separation due to strong $\:\overrightarrow{{E}_{\text{field}}}$ at the p^+^-FLG/n^–^-ZnO NWs heterojunction, and bias-dependent drift velocity, which ultimately contribute to the *J*_light_ in the external circuit.

**Fig. 11:**
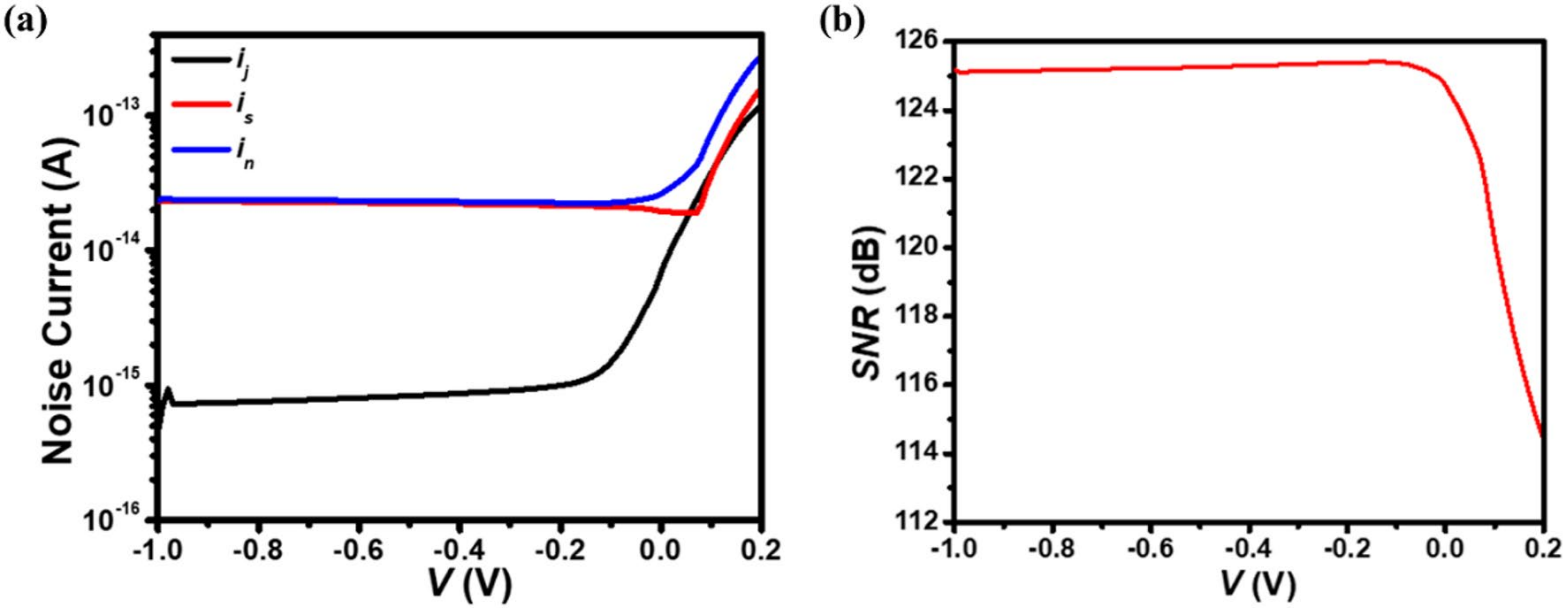
The simulated plot of **(a)** noise current components and **(b)** *SNR* for p^+^-FLG/n^–^-ZnO NWs heterojunction-based UV photodetector under applied reverse biasing conditions.

Moreover, the total noise current $\:{i}_{\text{n}}\left(\text{V}\right)\text{=}\sqrt{\frac{\text{4}{k}_{\text{B}}{T}}{RA}\text{+2}{q}\left({{J}_{\text{dark}}\text{+}J}_{\text{light}}\right){A}}$^40^ under the applied reverse bias is estimated to be 2.64 × 10^–14^ and 2.33 × 10^–14^ A under self-biasing and photoconductive (–0.5 V) mode of operation, and is better than the earlier stated values^35^. The former part $\:\sqrt{\frac{\text{4}{k}_{\text{B}}{T}}{RA}}$ represents the Johnson noise current (*i*_j_), and the latter part$\:\sqrt{\text{2}{q}\left({J}_{\text{dark}}\text{+}{J}_{\text{light}}\right){A}}$ denotes the shot noise current (*i*_s_). The comparison of *i*_n_, *i*_J_, and *i*_s_ under the applied reverse bias voltage is depicted in Fig. 11(a). It can be seen that the shot noise current increases with the applied reverse bias voltage because of the large value of *J*_dark_ and is the major source of noise in the proposed photodetector. On the other hand, the contribution to the total noise current due to the Johnson noise current is small as compared to the shot noise current signifying that the unwanted thermal generation-recombination current component is proficiently filtered out. The variation of signal-to-noise ratio $\:\left({SNR}\left(\text{dB}\right)\text{=10lo}{\text{g}}_{\text{10}}\left(\frac{{QE}_{\text{ext}}{P}_{\text{in}}{\lambda}_{\text{c}}}{\text{2.48}{i}_{\text{n}}\left(\text{V}\right)}\right)\right)$^40^ at center wavelength with voltage is shown in Fig. 11(b) and is estimated to be 123.7 and 125.30 dB at 0 and −0.5 V, respectively. The decrease in *SNR* with positive bias voltage is due to increased noise current. Table 2 provides a comparative summary of optoelectronic characteristics for the present work and selected previous studies^2,22,35,53–55,84,93,98^, measured at the center wavelength under 1 W/cm^2^ illumination intensity. The table highlights the significant advancements in the proposed UV photodetector, showcasing improvements in key performance metrics.

**Table 2:** Comparative analysis of the performance of the proposed photodetector with earlier reported photodetectors.

Device structure	*J*_light_/*J*_dark_	τ_*r*_ / τ_f_ (ns)	Quantum efficiency (%)	Responsivity (A/W)	D^*^ (Jones)	*NEP* (W)	λ (nm)	Ref.
WSe_2_/ZnO heterojunction	–	496 × 10^3^ / 515 × 10^3^	18@0 V	0.045@0 V	~10^9^@0 V	–	300	^2^
ZnO/Ga_2_O_3_ heterojunction	416	2.7 × 10^9^ / 5 × 10^9^	–	–	–	–	300	^22^
Graphene/ZnO/n-Si photodetector	10^4^	0.28 / 0.54	–	0.5	3.9 × 10^13^	–	488	^35^
ZnO NWs/graphene**/**Cu_2_O heterojunction	–	6 × 10^6^ / 6 × 10^6^	–	0.0212@365 nm, 0.0171@450 nm	–	–	365 and 450	^53^
Graphene/ZnO NWs heterojunction	–	28 × 10^6^ / 25 × 10^6^		3.5 × 10^− 6^	–	–	325	^54^
ZnO NWs/TiO_2_-GO	–	0.89 × 10^9^ / 1.66 × 10^9^	–	13.52	–	–	365	^55^
p-Si/graphene/n-ZnO photodetector	10.71	1.02 × 10^9^ / 0.34 × 10^9^	–	–	–	–	365	^84^
Graphene QDs/ZnO nanorods/GaN	–	0.1 × 10^9^ / 0.12 × 10^9^	–	0.034@10 V	~ 10^12^	–	365	^93^
Graphene/Si	–	–	–	0.18	–	–	390	^98^
p^+^-FLG/n^–^-ZnO NWs	2.5 × 10^5^ @ 0 V	0.26 / 0.26 @ 0 V	44.1 @ 0 V	0.12@0 V	1.9 × 10^9^@ 0 V	5.6 × 10^–14^@ 0 V	350	This work
	16.25 @ −0.5 V	0.16 / 0.16 @ −0.5 V	56 @ −0.5 V	0.2@ –0.5 V	2.4 × 10^9^@ −0.5 V	4.4 × 10^− 14^ @ −0.5 V		

The proposed photodetector demonstrates superior photocurrent responsivity (up to 0.26 A/W), fast response times (as low as 0.26 ns), and exceptional on/off ratios (reaching 2.5 × 10^5^). These improvements are attributed to advanced device engineering techniques, such as core-shell structures and the incorporation of 2D graphene materials. Furthermore, the enhanced performance can be attributed to the combined effect of high carrier mobility in the graphene layers and the existence of a strong electric field (136.82 kV/cm) at p^+^-FLG/n^–^-ZnO NWs heterojunction. This strong electric field facilitates the efficient separation of photoexcited carriers, contributing significantly to the photodetector’s overall performance. These values far exceed those of previous ZnO-based photodetectors, such as WSe₂/ZnO, which exhibited slower response times in the microsecond range and lower photocurrent responsivity^2^. Additionally, the photodetector achieves an *NEP* of 4.4 × 10^–14^ W, improving detection sensitivity and *SNR*. Unlike many existing ZnO-based photodetectors that require higher external biasing for optimal performance, the proposed device operates efficiently in both self-powered and photoconductive modes, making it highly suitable for low-power applications.

This remarkable performance paves the way for exciting real-world applications of ZnO NWs photodetectors. For example, the high UV sensitivity and fast response make them ideal for environmental monitoring of UV radiation levels, enabling portable wearable devices that can provide real-time alerts about harmful exposure. Their high speeds also open up uses in optical communications like Li-Fi and visible light communication (VLC) systems integrated into smart lighting for simultaneous illumination and data transmission. Additionally, the large surface area of the nanowires allows functionalization for selective biological/chemical sensing by tailoring the surface chemistry, paving the way for compact real-time sensors for biomolecule/gas detection. While continuing to enhance performance metrics, a key focus area is integrating these photodetectors into practical systems across environmental, communication, and sensing applications.

Moreover, while this study primarily focused on evaluating the optoelectronic characteristics of the photodetector under room temperature, it is important to note that the current simulations did not explicitly account for strain effects. Strain can significantly affect devices by modifying the bandgap, altering carrier mobility, and inducing piezoelectric effects. These changes are particularly relevant in photodetectors, where strain modulation has been shown to enhance optoelectronic properties. For instance, in the study by Liu et al.^99^, tensile strain modulation through the piezoelectric effect in a graphene/ZnO NW heterostructure led to improved photodetector performance by enhancing photogenerated electron injection and optimizing the material’s response to light. While strain effects were not considered in the present simulations, incorporating these effects could provide additional insights into the behavior of the proposed photodetector. Future work will aim to include strain to evaluate its impact on device performance. However, the current results still provide a valuable foundation for understanding the devices under unstrained conditions, often the early-stage research’s initial focus.

## Conclusions

In summary, a highly efficient p^+^-FLG and n^–^-ZnO NWs-based heterojunction photodetector at 300 K under the UV spectrum regime is presented. The photodetector can work in self-biasing conditions in addition to a photoconductive mode of operation. The Silvaco TCAD simulation software is used to study the optoelectronic parameters which are further validated by the drift-diffusion approach based on analytical expressions. The enhancement in photocurrent density is achieved under the reverse bias attributed to effective carrier generation at the hetero-interface of p^+^-FLG and n^–^- ZnO NWs. The p^+^-FLG/n^–^-ZnO NWs photodetector demonstrates sharp and rapid photoswitching times of 0.16 ns at −0.5 V. The $\:{R}_{\text{i}}^{\text{ext}}$, *QE*_ext_, D^*^, and *NEP* of 0.2 A/W, 56%, 2.4 × 10^9^ Jones, and 4.4 × 10^–14^ W, respectively, are obtained for the proposed photodetector. Moreover, the photodetector shows a maximum value of *QE*_ext_ of 68% at − 5.0 V. This is attributed to the better absorption by ZnO NWs in the UV regime and the presence of strong built-in electric field p^+^-FLG/n^–^-ZnO NWs heterojunction which quickly separates the photo-induced carriers to the electrodes resulting in the photocurrent.

In the near future, it is planned to focus on optimizing the graphene/ZnO NWs hetero-interface, exploring different hybrid architectures (e.g., core-shell, radial heterostructures), and investigating scalable fabrication techniques for large-area, flexible, and cost-effective devices. Additionally, future work will incorporate strain effects to better understand their impact on device performance. The integration of graphene with other materials could enable self-biasing and flexible UV photodetectors, opening up new avenues for wearable and internet-of-things (IoT) applications in areas such as personal UV monitoring, monitoring, biomedical sensing, and secure optical communications. With continued advancements in material synthesis, device engineering, and system integration, UV photodetectors employing graphene/ZnO NWs show immense potential for the development of next-generation self-driving, highly efficient, and cost-effective broadband photodetectors with outstanding optoelectronic properties.

## Electronic supplementary material

Below is the link to the electronic supplementary material.

Supplementary Material 1

## Data Availability

All data generated or analyzed during this study are included in this article. None Declare.
