# Author Correction: Design and TCAD analysis of few-layer graphene/ZnO nanowires heterojunction-based photodetector in UV spectral region

**DOI:** 10.1038/s41598-025-97341-4

**Published:** 2025-04-15

**Authors:** Shonak Bansal, Sandeep Kumar, Arpit Jain, Vinita Rohilla, Krishna Prakash, Anupma Gupta, Tanweer Ali, Abdulmajeed M. Alenezi, Mohamed Shabiul Islam, Mohamed S. Soliman, Mohammad Tariqul Islam

**Affiliations:** 1 Department of Electronics and Communication Engineering, Chandigarh University, Gharuan, Punjab India; 2 School of Computer Science and Artificial Intelligence, SR University, Warangal, India; 3 Department of Computer Science and Engineering, Koneru Lakshmaiah Education Foundation Vadeshawaram, A.P Guntur, India; 4 Department of Computer Science and Engineering, Maharaja Surajmal Institute of Technology, C-4 Janakpuri, New Delhi, India; 5 Department of Electronics and Communication, NRI Institute of Technology, Agripalli, Eluru, 521212 AP India; 6 Department of Electronics and Communication Engineering, Saveetha School of Engineering, Saveetha Institute of Medical and Technical Sciences, Thandalam, Chennai, Tamilnadu India; 7 Department of Electronics and Communication Engineering, Manipal Institute of Technology, Manipal Academy of Higher Education, Manipal, 576104 India; 8 Department of Electrical Engineering, Faculty of Engineering, Islamic University of Madinah, Madinah, 42351 Saudi Arabia; 9 Faculty of Engineering (FOE), Multimedia University (MMU), Cyberjaya, 63100 Selangor Malaysia; 10 Department of Electrical Engineering, College of Engineering, Taif University, Taif, 21944 Saudi Arabia; 11 Department of Electrical, Electronic and Systems Engineering, Faculty of Engineering and Built Environment, 43600 UKM Bangi, Selangor Bangi, Malaysia

Correction to: *Scientific Reports* 10.1038/s41598-025-92596-3, published online 05 March 2025

The original version of this Article contained an error in the Results and Discussions section where the in-text reference to Fig. 10 was incorrect.

“On the other hand, the *NEP* decreases exponentially from 3.6 × 10^–14^ to 5.6 × 10^–14^ W with applied reverse bias voltage varying from –5.0 to 0 V according to the Fig. 10. expression ${\text{A}}_{2}+{\text{A}}_{3}{e}^{\left(-\frac{\text{V}}{{t}_{2}}\right)}$ with *R*^2^ = 0.94. Here *A*_2_, *A*_3_, and *t*_2_ are the fitted parameters.”

now reads:

“On the other hand, the *NEP* decreases exponentially from 3.6 × 10^–14^ to 5.6 × 10^–14^ W with applied reverse bias voltage varying from –5.0 to 0 V according to the expression ${\text{A}}_{2}+{\text{A}}_{3}{e}^{\left(-\frac{\text{V}}{{t}_{2}}\right)}$ with *R*^2^ = 0.94. Here *A*_2_, *A*_3_, and *t*_2_ are the fitted parameters.”

The original Article has been corrected.

