# Supplementary material for: Design and TCAD analysis of few-layer graphene/ZnO nanowires heterojunction-based photodetector in UV spectral region
Source: Sci Rep. 2025 Mar 5;15:7762. doi: 10.1038/s41598-025-92596-3 (PMC11882988; doi:10.1038/s41598-025-92596-3)
Supplement: Supplementary file 1 — Supplementary Material 1 [file 41598_2025_92596_MOESM1_ESM.docx]

Electronic Supplementary Material

Design and TCAD analysis of few-layer graphene/ZnO nanowires heterojunction-based photodetector in UV spectral region

Shonak Bansal^1*^, Sandeep Kumar^2^, Arpit Jain^3^, Vinita Rohilla^4^, Krishna Prakash^5*^, Anupma Gupta^1,6^, Tanweer Ali^7^, Abdulmajeed M. Alenezi^8^, Mohamed Shabiul Islam^9^*, Mohamed S. Soliman^10^, and Mohammad Tariqul Islam^11*^

^1^Department of Electronics and Communication Engineering, Chandigarh University, Gharuan, Punjab, India

^2^School of Computer Science and Artificial Intelligence, SR University, Warangal, India

^3^Department of Computer Science and Engineering Koneru Lakshmaiah Education Foundation Vadeshawaram, A.P., India

^4^Department of Computer Science and Engineering, Maharaja Surajmal Institute of Technology, C-4, Janakpuri, New Delhi

^5^Department of Electronics and Communication, NRI Institute of Technology, Agripalli, Eluru-521212, AP, India

^6^Department of Electronics and Communication Engineering, Saveetha School of Engineering, Saveetha Institute of Medical and Technical Sciences, Thandalam, Chennai, Tamilnadu, India

^7^Department of Electronics and Communication Engineering, Manipal Institute of Technology, Manipal Academy of Higher Education, Manipal 576104, India.

^8^Department of Electrical Engineering, Faculty of Engineering, Islamic University of Madinah, Madinah 42351, Saudi Arabia

^9^Faculty of Engineering (FOE), Multimedia University (MMU), 63100 Cyberjaya, Selangor, Malaysia.

^10^Department of Electrical Engineering, College of Engineering, Taif University, Taif 21944, Saudi Arabia.

^11^Department of Electrical, Electronic and Systems Engineering, Faculty of Engineering and Built Environment, 43600 UKM Bangi, Selangor, Malaysia

^*^Corresponding author’s Email: [shonakk@gmail.com](mailto:shonakk@gmail.com), [k_krishna2k7@yahoo.co.in](mailto:k_krishna2k7@yahoo.co.in), [shabiul.islam@mmu.edu](mailto:shabiul.islam@mmu.edu), tariqul@ukm.edu.my

ORCiD ID: 0000-0002-6551-6011

**Device Design Optimization**

The design and optimization of the p⁺-FLG/n⁻-ZnO NWs-based UV photodetector were carried out to investigate how structural parameters influence overall device performance. To systematically compare performance, simulations were conducted for ZnO NW arrays with three different configurations: 2×2, 4×4, and 5×5. These simulations provided valuable insights into the impact of nanowire density on the overall functionality of the device. The results revealed that increasing the nanowire density significantly enhances light absorption and charge carrier generation due to the larger active area and improved interaction with the built-in electric field at the heterojunction. For instance, the 5×5 configuration demonstrated superior performance, with a 56% higher photocurrent responsivity compared to the 2×2 array (Fig. S1). However, a trade-off exists as higher nanowire densities can lead to fabrication complexities and inter-wire shadowing effects.

While simulations focused on nanowire density, other structural parameters including the ZnO NWs dimensions (200 nm diameter, 1500 nm length, and 20 nm spacing)-and material properties were optimized based on previously reported theoretical and experimental works. The high aspect ratio (7.5:1) of the ZnO NWs enhanced UV light absorption, while the inter-wire spacing minimized recombination and maintained structural stability. Additionally, a thin 10 nm ZnO seed layer was incorporated to ensure uniform nanowire alignment and reduce defects at the heterojunction interface.

Through this comparative analysis of ZnO NW array configurations, the 5×5 configuration was identified as the optimal design, balancing high performance with practical manufacturability. These findings clarify the critical role of nanowire density in optimizing device performance while highlighting specific factors such as light absorption, charge carrier generation, and inter-wire interactions that influence the photodetector’s efficiency.


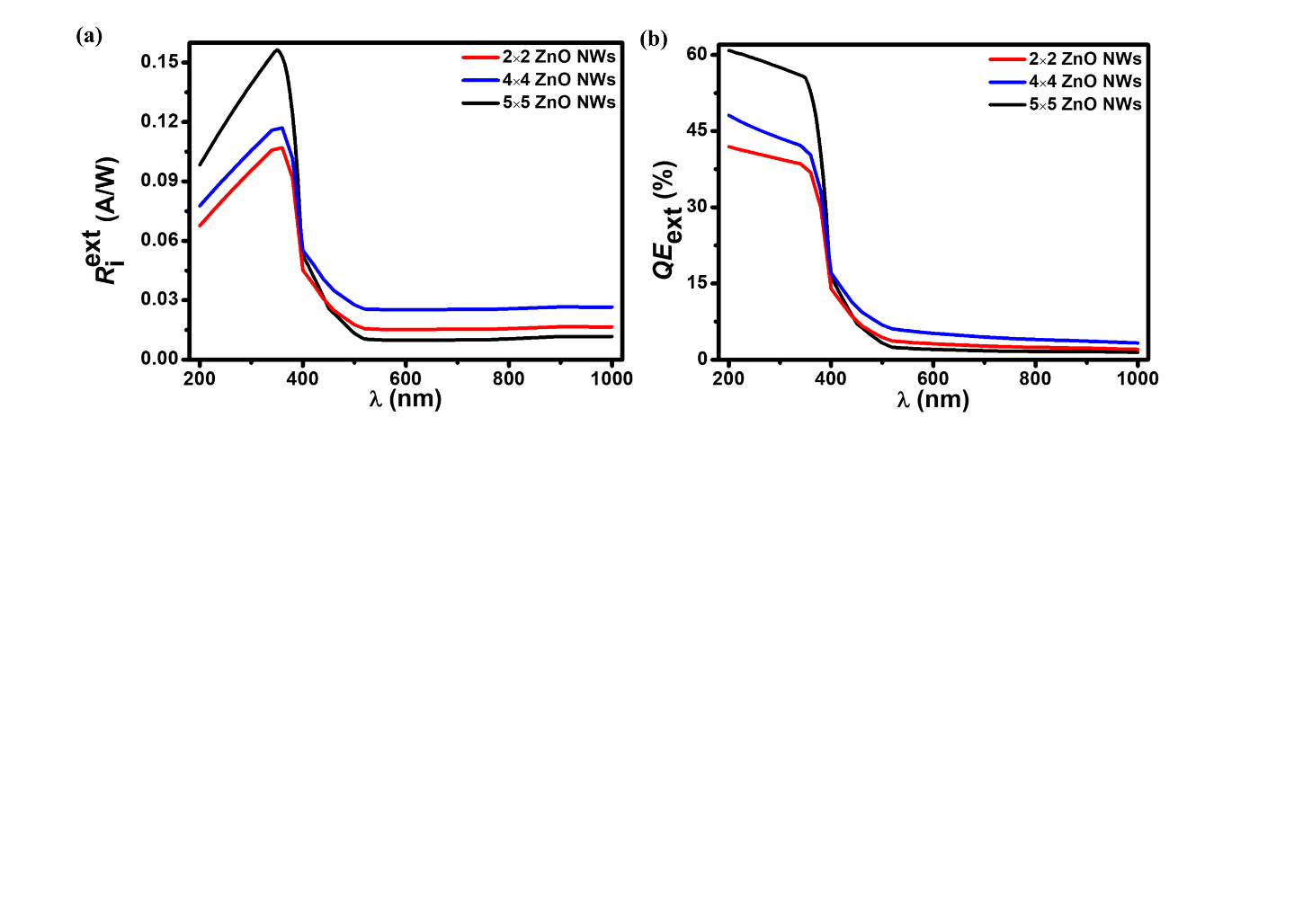


**Figure S1.** The simulated optical characteristics of the p^+^-FLG/n^–^-ZnO NWs photodetector under –0.5 V with 1 W/cm^2^ illumination intensity at 350 nm, evaluated for three ZnO NWs array configurations: 2×2, 4×4, and 5×5. **(a)** $\text{R}_{i}^{\text{ext}}$-λ characteristics. **(b)** *QE*_ext_-λ characteristics.


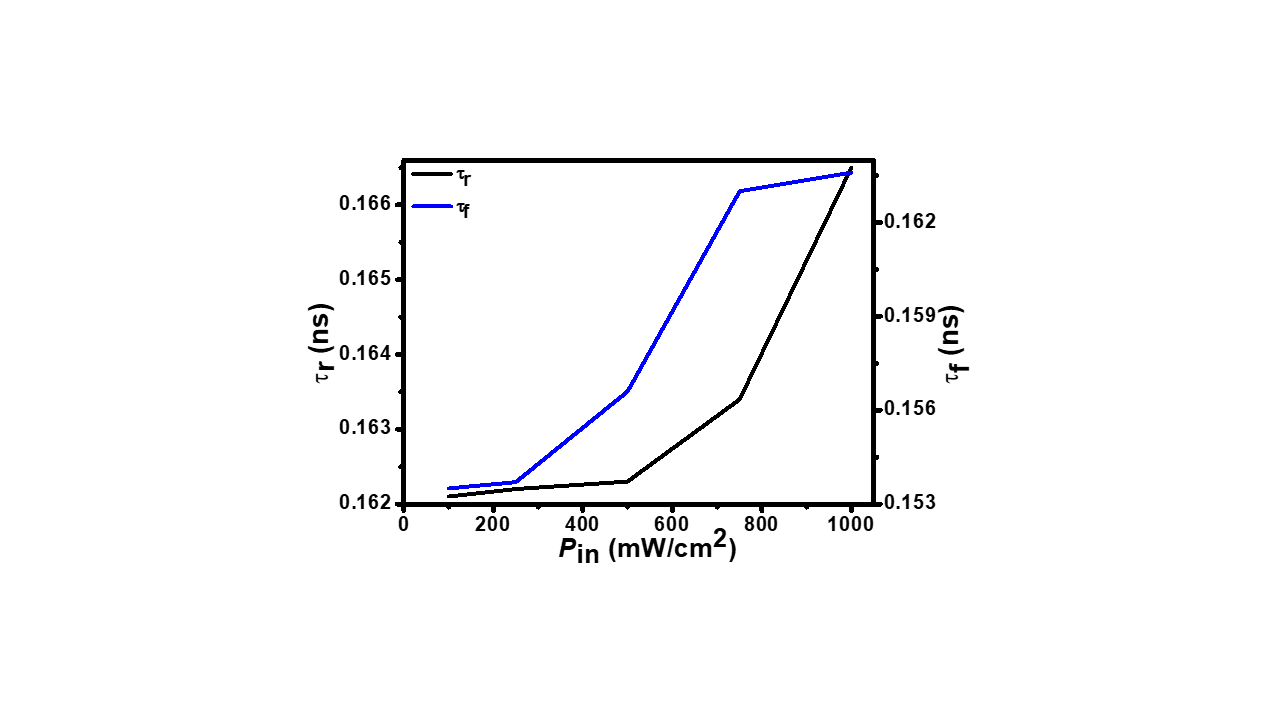


**Figure S2.** The variation of rise time (τ_r_) and fall time (τ_f_) with power intensities at 350 nm wavelength under the bias of –0.5 V.
